# Engineered Probiotics Mitigate Gut Barrier Dysfunction Induced by Nanoplastics

**DOI:** 10.1002/advs.202417283

**Published:** 2025-04-01

**Authors:** Wenxin Chen, Qiyan Guo, Hong Li, Xue Chi, Xiang Ma, Yanqiong Tang, Quanfeng Liang, Zhu Liu, Yong Liu, Juanjuan Li

**Affiliations:** ^1^ School of Life and Health Sciences Hainan Province Key Laboratory of One Health Collaborative Innovation Center of One Health Hainan University Haikou 570228 China; ^2^ School of Chemistry and Chemical Engineering Hainan University Haikou 570228 China; ^3^ Faculty of Animal Science and Technology Key Laboratory of Animal Nutrition and Feed Science of Yunnan Province Yunnan Agricultural University Kunming 650201 China; ^4^ State Key Laboratory of Microbial Technology Shandong University Jinan 250100 China

**Keywords:** engineered probiotics, gut barrier, intestinal flora, nanoplactics

## Abstract

Micro‐ and nanoplastics, particularly those derived from food‐contact materials like polyethylene terephthalate (PET), can damage gut barriers, making the gastrointestinal system more vulnerable to inflammation and infections. Here, a probiotic‐based drug delivery system (EcN_T_@L) is devised to mitigate nanoplastics‐induced gut barrier dysfunction by modulating gut immunity and microbiota. *Escherichia coli* Nissle 1917 (EcN) is genetically engineered to produce transforming growth factor‐β (TGF‐β) and then modified with an Eudragit L100‐55 coating. This engineered probiotic acts as an in vivo “drug factory”, exerting anti‐inflammatory and immune‐regulatory effects, with improved retention and bioavailability in the gastrointestinal tract. EcN_T_@L effectively protects Caco‐2 cells from inflammation and infections induced by nano PET, primarily by activating the NF‐κB signaling pathway. Besides, EcN_T_@L demonstrates superior in vivo therapeutic efficacy in treating gastrointestinal infection caused by the combined presence of nano PET and *Salmonella*, outperforming commercial antibiotics due to its ability to modulate immune responses and gut microbiota. This study highlights the potential of probiotic‐based drug delivery systems in addressing nanoplastics‐induced gut dysfunctions, offering a promising strategy for mitigating the environmental impact of micro‐ and nanoplastics.

## Graphical abstract

Intestinal barrier stability is vital for gut health. Plastic polyethylene terephthalate (PET) disrupts this barrier, causing inflammation and infections. This study encodes transforming growth factor‐β into EcN and modifies its surface with pH‐responsive polymer (EcN_T_@L), creating an oral drug delivery system to repair nano PET‐induced intestinal damage.

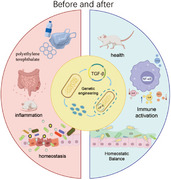

## 1 Introduction

Micro‐ and nanoplastics are resilient to environmental conditions and can easily enter the food chain. Once ingested, they accumulate in the gastrointestinal tract, where they can damage the intestinal barrier, leading to increased barrier permeability, microbiota alteration, oxidative stress, inflammation, and other adverse effects.^[^ ^1^, ^2^, ^3^, ^4^, ^5^ ^]^ As an emerging contaminant, the effective treatment of gut disorders caused by micro/nanoplastics is still being developed. Restoring the damaged gut barrier is crucial for preventing nano/microplastics‐induced gut inflammation and infections.^[^ ^6^, ^7^ ^]^ Common strategies for gut barrier repair include applying probiotics and prebiotics,^[^ ^8^, ^9^, ^10^ ^]^ anti‐inflammatory drugs (e.g., mesalazine),^[^ ^11^ ^]^ tight junction modulators (e.g., larazotide acetate),^[^ ^12^ ^]^ and mucosal immunumodulators.^[^ ^13^ ^]^ Among various strategies, probiotics are particularly effective due to their intrinsic activity to interact with the gut barrier network and excellent biocompatibility. Besides, probiotics have the potential for multifunctionality in synergistically modulating both the immune microenvironment and gut microbiota.^[^ ^14^ ^]^ Despite the growing consensus that probiotics offer promise in reducing micro/nanoplastic toxicity to the gastrointestinal system, there are, to our knowledge, few studies that demonstrate this effect.^[^ ^15^, ^16^ ^]^

Conventional probiotic therapies face two major challenges: a) limited oral bioavailability due to poor survival in the harsh gastric and intestinal environment,^[^ ^17^, ^18^ ^]^ and b) the lack of designed multifunctionality for on‐demand drug delivery.^[^ ^19^, ^20^ ^]^ Single‐cell encapsulation of probiotics within a protective coating allows enhanced oral bioavailability and may impart new features to the bacterial cells in treating gastrointestinal diseases and tumors.^[^ ^21^, ^22^, ^23^, ^24^, ^25^ ^]^ Although therapeutic drugs can be incorporated onto the surface of probiotics for targeted delivery, this strategy suffers from poor stability and uncontrolled drug release profiles.^[^ ^26^, ^27^ ^]^ Bacteria‐based “drug factories” enabled by genetic engineering has emerged as a promising alternative for delivering peptides or proteins while preserving the inherent biological activity of the bacteria.^[^ ^28^ ^]^ Various immunotherapeutic agents,^[^ ^29^, ^30^, ^31^, ^32^ ^]^ such as α‐PD‐1, have been successfully targeted to specific sites for precise regulation and effective activation.^[^ ^33^ ^]^ However, combining the two advantages, i.e., improved bioavailability and precise drug delivery, remains challenging. Achieving this would, as expected, enhance the viability of probiotics in the gastrointestinal environment and enable targeted drug production at the desired site. To do this, we introduced a doubly engineered probiotic therapeutic, EcNT@L, to explore the potential of probiotics in alleviating nanoplastics‐induced gut barrier dysfunction.

Herein, we developed a doubly engineered probiotic therapeutic, EcN_T_@L, to explore the potential of probiotics in alleviating nanoplastics‐induced gut barrier dysfunction. We engineered *Escherichia coli* Nissle 1917 (EcN) to produce the anti‐inflammatory transforming growth factor‐β (TGF‐β), and then encapsulated them within a pH‐responsive polymer, Eudragit L100‐55.^[^ ^34^ ^]^ Compared to unmodified EcN, EcN_T_@L exhibited enhanced viability and retention in the gut, along with in situ synthesis and release of TGF‐β. The engineered probiotics effectively reduced the inflammatory response in Caco‐2 cells induced by nano PET, preventing *Salmonella* colonization aggravated by nanoplastics. Besides, they significantly eliminated *Salmonella* within infected cells. By activating the NF‐κB pathway and modulating gut microbiota, EcN_T_@L demonstrated superior efficacy over conventional antibiotics in combating nano PET‐associated *Salmonella* infections in murine models.

## 2 Results

### 2.1 Construction of EcN_T_@L

**Figure 1:**
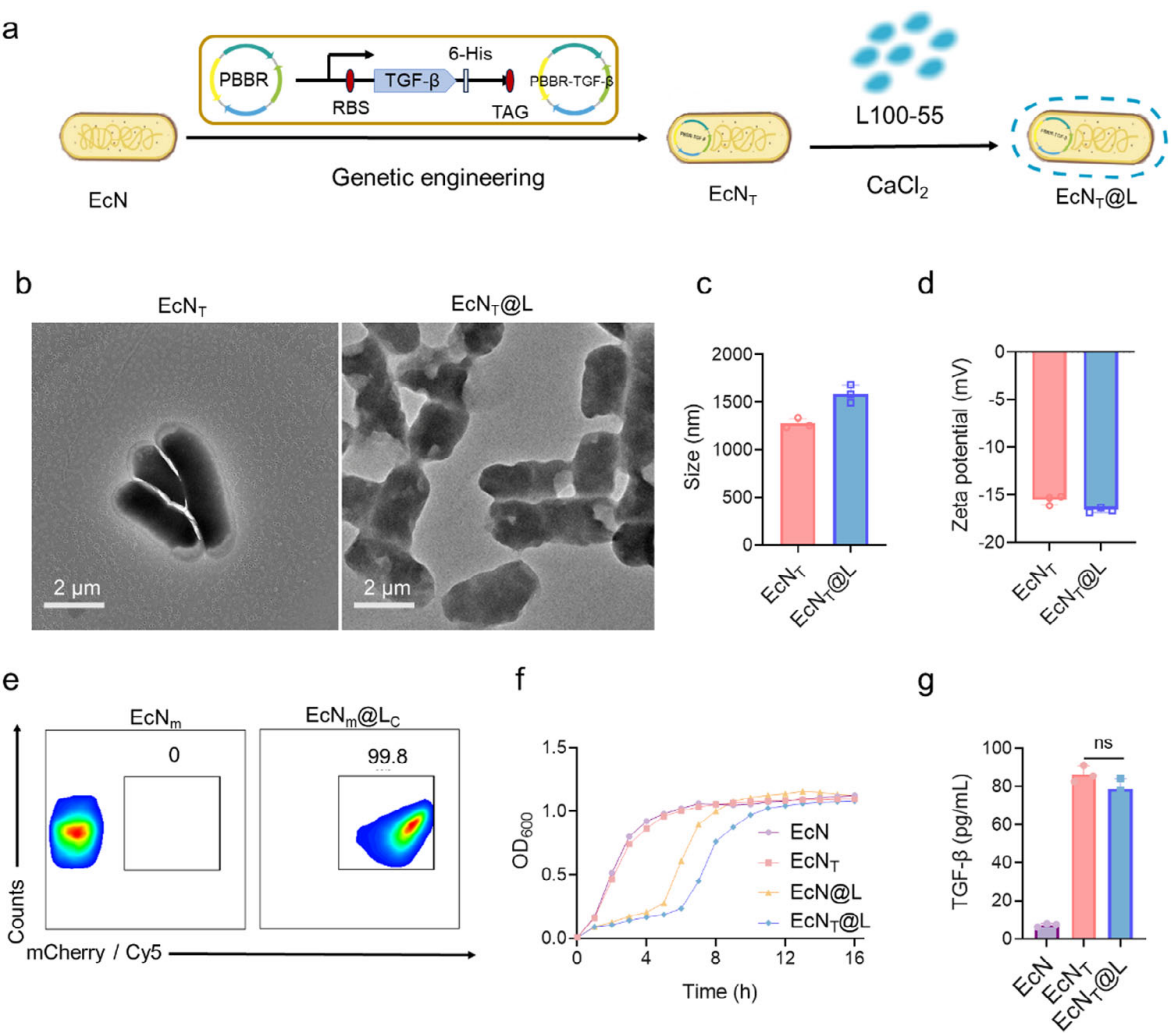
Preparation and characterization of EcN_T_@L. a) Synthesis of EcN_T_@L. L100‐55 capped on EcN_T_ by using Ca^2+^ crosslink. b) TEM images of EcN_T_ and EcN_T_@L. Scale bar: 2 µm. c,d) Sizes distribution (c) and zeta potential (d) of EcN_T_ and EcN_T_@L. e) Flow cytometry assay of EcN_m_ and EcN_m_@L. Cy5 was used to label L100‐55. f) The growth curve of EcN, EcN_T_, EcN@L, and EcN_T_@L in LB medium at 37 °C. g) The concentration of TGF‐β in supernatant of EcN, EcN_T_, and EcN_T_@L post a 12 h incubation in LB medium.

Several probiotics, including EcN, *Bacillus subtilis*, and *Lactobacillus reuteri*, have been used to treat gastrointestinal infections.^[^ ^35^ ^]^ Among them, EcN exhibits antagonistic effects against pathogens such as *Salmonella typhimurium* and *Clostridium nori*, stimulates immune responses, and helps maintain intestinal flora homeostasis. Importantly, compared to other probiotics, EcN offers a model host for exogenous gene expression due to its well‐characterized genetic background and simple operational requirements. Therefore, we transferred a pBBRMCS‐2 vector containing a TGF‐β gene that equipped into EcN, resulting in an EcN variant expressing 15.8 kD TGF‐β (EcN_T_). Since the plasmids expressing mCherry or TGF‐β conferred kanamycin resistance, EcN_m_ and EcN_T_ could be easily distinguished from other microorganisms on agar plates containing kanamycin (50 µg mL^−1^). We next decorated EcN_T_ with a pH responsive L100‐55 coating (**Figure** **1**a; Figure S1, Supporting Information). The modification of single probiotic cells with L100‐55 was achieved under pH 5.5 in the presence of Ca^2+^, harnessing its self‐crosslinking property.^[^ ^36^, ^37^ ^]^ L100‐55 formed a 163.1 ± 38.5 nm shell on single EcN_T_ immediately after adding a proper amount of HCl (Figure 1b), with negligible cell toxicity to EcN_T_ (Figure S2, Supporting Information). Surface coating on EcN_T_ increased the average size of bacteria from 618.8 ± 42.0 nm × 1403.0 ± 124.7 nm to 846.9 ± 32.8 nm × 1582.6 ± 23.0 nm (Figure 1c), and decreased their ζ‐potential from −15.5 to −16.6 mV (Figure 1d). Compared with unmodified EcN_T_, EcN_T_@L displayed a rough surface morphology (Figure S3, Supporting Information). Fourier transform infrared spectrometer analysis revealed peaks at 1388, 1475, and 2989 cm^−1^ in the spectrum of EcN_T_@L, but not in EcN_T_ (Figure S4, Supporting Information), which were assigned to the stretching and vibration peaks of ─NH_2_ in L100‐55. Flow cytometry analysis indicated a 99.8% encapsulation efficiency of EcN_T_@L using fluorescence labeled EcN (EcN‐mCherry, EcN_m_) and L100‐55 (Cy5‐conjugated L100‐55, L_c_, Figure 1e).

We investigated the impact of L100‐55 coating on the proliferation and TGF‐β secreting ability of EcN_T_.^[^ ^38^ ^]^ Compared to EcN and EcN_T_, EcN_T_@L showed a delayed growth during the initial 2.5 h, but reached a similar growth rate after 6 h post incubation (Figure 1f), indicating the favorable safety of L100‐55 coating. The expressing level of TGF‐β in EcN_T_ and EcN_T_@L post 24 h incubation was 86.4 ± 5.1 and 78.8 ± 4.3 pg mL^−1^, respectively (Figure 1g), suggesting that L100‐55 did not impede TGF‐β expression by EcN_T_. Together, we successfully fabricated EcN_T_@L with the ability to secrete TGF‐β, demonstrating high encapsulation efficiency and excellent biosafety.

### 2.2 L100‐55 Improved the Bioavailability of EcN_T_@L

**Figure 2:**
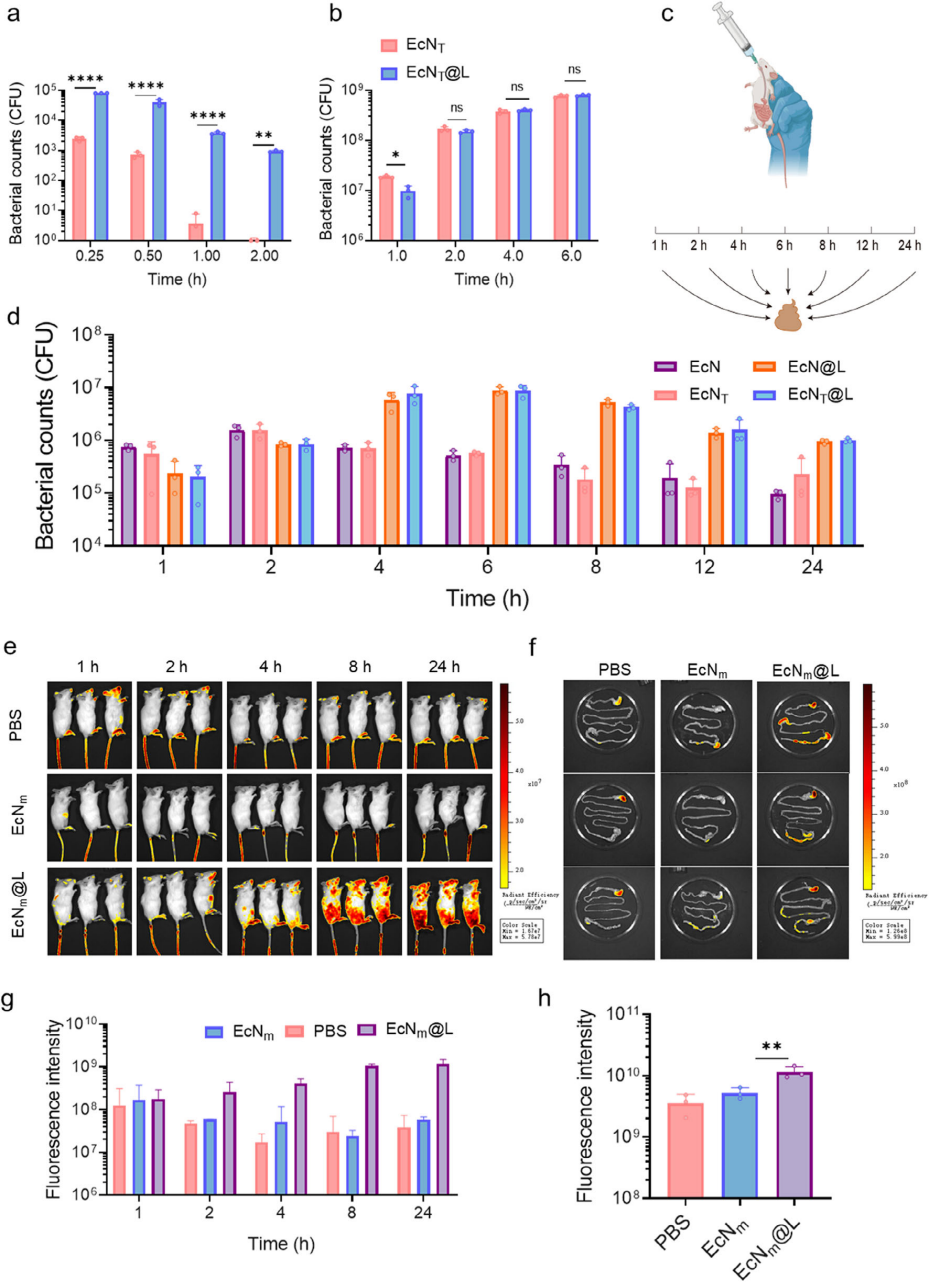
L100‐55 improved bioavaibility and gut retention of EcN_T_. a,b) Bacterial count of EcN_T_ and EcN_T_@L in SGF (a) and SIF (b) post incubation for different time. c,d) Experimental design (c) and bacterial counts (d) in feces of mice treated with EcN, EcN_T_, EcN@L, and EcN_T_@L. e,g) Fluorescence images (e) and mean fluorescence intensity (MFI) (g) of mice post oral administration of EcN_m_ or EcN_m_@L for different time point. f,h) Fluorescence images (f) and MFI (h) of gastrointestinal tract post oral administration of EcNm or EcNm@L. *n* = 3, *p ≤ 0.05, **p ≤ 0.01, ****p ≤ 0.0001.

The digestive gastrointestinal system, particularly gastric acid, severely reduced the survival rate of probiotics.^[^ ^39^ ^]^ L100‐55, commercially used in the capsule industry, showed superior protective ability against gastric fluid due to its pH‐responsive properties.^[^ ^40^ ^]^ To assess its effect on probiotic bioavailability, we incubated EcN_T_ and EcN_T_@L in simulated intestinal fluid (SIF) and simulated gastric fluid (SGF), and then counted the viable EcN_T_ cells. The L100‐55 coating increased the survival rate of EcN_T_ in SGF by 20‐fold compared to uncoated EcN_T_ at 15 min post incubation (**Figure** **2**a), indicating the protective effect of L100‐55 on EcN_T_ in harsh environments. Besides, while L100‐55 inhibited the growth of EcN_T_ at the initial stage, it did not significantly impede bacterial growth in SIF after that (Figure 2b), due to the dissolvable nature of L100‐55 in neutral environment.^[^ ^41^ ^]^ In SIF, the L100‐55 shell detached from the bacterial surface after 2 h of incubation (Figure S5, Supporting Information), confirming the decoating behavior of L100‐55. Thus, L100‐55 effectively protected the probiotics without compromising their viability or functionality.

To assess the oral delivery performance of EcN_T_@L, we administered equal amounts of EcN, EcN@L, EcN_T_, and EcN_T_@L to mice and subsequently quantified the number of EcN and EcN_T_ in feces at various time intervals (Figure 2c). The hemolysis ratio for all bacterial agents remained below 10% (Figure S6, Supporting Information), and no significant inflammation was observed in the mice following the oral administration of EcN_T_@L (Figures S7 and S8, Supporting Information). Notably, at 4, 6, 8, 12, and 24 h post administration, the bacterial counts in the EcN@L and EcN_T_@L groups were found to be 50 times greater than those in the EcN and EcN_T_ groups (Figure 2d), indicating that the L100‐55 coating significantly enhanced the colonization capability of the probiotics within the gastrointestinal tract. To visualize the distribution and colonization of the probiotics, mice were administered phosphate‐buffered saline (PBS), EcN_m_, and EcN_m_@L (10^9^ CFU), followed by imaging with an in vivo imaging system (IVIS). Mice treated with EcN_m_@L exhibited the highest fluorescence intensity at 6 h post administration, which remained stable throughout a 24 h observation period (Figure 2e,g). EcN underwent a decoating process from the L100‐55 shell after reaching the intestine, taking ≈2.5 h to release the encapsulated cells. These cells then colonized the gut and proliferated until reaching the plateau stage, leading to an increase in fluorescence intensity between 8 and 24 h post administration.^[^ ^42^ ^]^ In contrast, fluorescence levels were markedly lower in mice treated with PBS and EcN_m_, suggesting that the L100‐55 coating effectively promoted the viability and retention of EcN. The average fluorescence intensity in the gastrointestinal tract of mice treated with EcN_m_@L was estimated to be fivefold higher than that of mice treated with EcN_m_ (Figure 2f,h), further underscoring the enhanced colonization ability conferred by the L100‐55 coating, the fluorescence signals in the stomach was attributed to the partially detached L100‐55. Besides, quantitative analysis indicated that in comparison to EcN_m_, EcN_m_@L exhibited a significantly higher level of living cells in the small intestine, colon, and cecum of mice (Figure S9, Supporting Information). Consequently, L100‐55 significantly improved the survival rate of probiotics during oral delivery, enhanced their bioavailability, and facilitated their colonization.

### 2.3 EcN_T_@L Suppressed Nano PET‐Associated Inflammation

**Figure 3:**
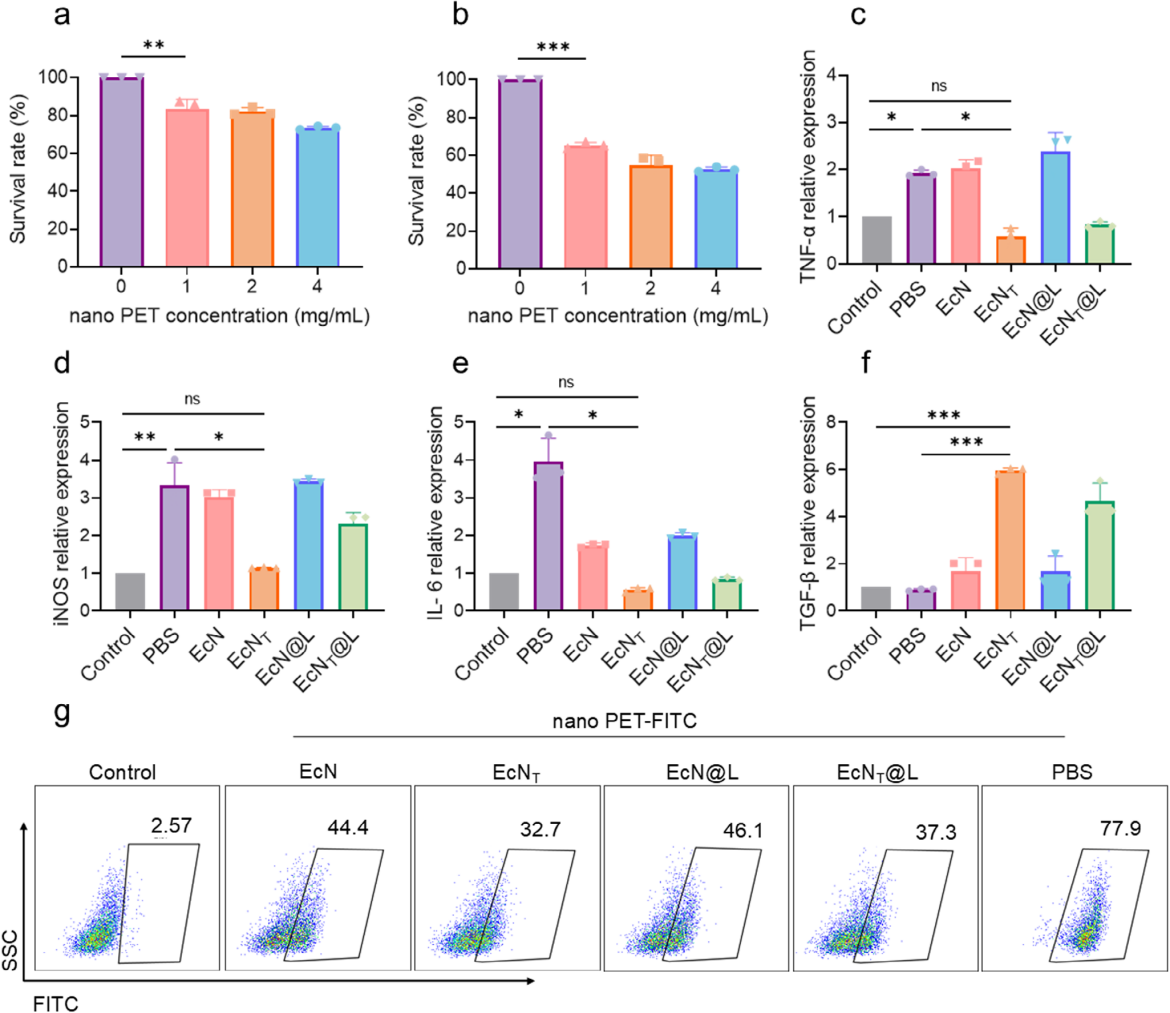
EcN_T_@L suppressed nano PET‐associated inflammation. a,b) Survival rate of Caco‐2 (a) and Raw264.7 (b) cells post treatment with different concentrations of nano PET. c–f) Relative expression of TNF‐α (c), iNOS (d), IL‐6 (e), and TGF‐β (f) in Caco‐2 cells treated with nano PET/PBS, nano PET/EcN, nano PET/EcN@L, nano PET/EcN_T_, and nano PET/EcN_T_@L. Cells without treatment were used as controls. g) Flow cytometry assay of Caco‐2 cells treated with nano PET‐FITC and different bacteria. *n* = 3, *p ≤ 0.05, **p ≤ 0.01, ***p ≤ 0.001.

Micro‐ and nanoplastics have been shown to accumulate in the gastrointestinal tract, leading to dysbiosis of gut microbiota and subsequent immune disorders.^[^ ^43^ ^]^ Probiotics, short chain fatty acid, and prebiotics have been proved to be effective for reliving gut inflammation.^[^ ^44^, ^45^, ^46^ ^]^ The extensive utilization of PET and the difficulty in its recycling have resulted in a considerable accumulation of nano PET waste in the global environment, inevitably causing its entry into the food chain.^[^ ^47^ ^]^ Both nano PET and nano PET‐FITC appeared as spherical nanoparticles (Figure S10, Supporting Information) and could disperse uniformly in aqueous solutions (Figure S11, Supporting Information). Nano PET and nano PET‐FITC possessed ζ‐potentials of −36.5 ± 0.3 and −28.9 ± 3.3 mV, respectively. Their average sizes were 120.3 ± 7.1 and 140.4 ± 4.8 nm (Figure S12, Supporting Information), respectively. We evaluated the impact of nano PET on cell viability. The cell viability of Caco‐2 and Raw264.7 cells decreased along with an increased concentration of nano PET (**Figure** **3**a,b), implying that high concentration of nano PET inhibited the cell proliferation. We then evaluated the penetrating ability of nano PET across gut barrier using a monolayer cell model. Compared to the control group, intense fluorescence of FITC was observed in the sublayer of cells treated with nano PET‐FITC (Figure S13, Supporting Information), illustrating that nano PET was able to cross the gut barrier. We next used FITC‐dextran to assess the damage of the gut barrier caused by nano PET. A high level of FITC‐dextran was detected in the sublayer of cells treated with nano PET (Figure S14, Supporting Information), indicating that nano PET damaged the gut barrier. We hypothesized that EcN_T_ could alleviate the damage of the intestinal epithelial structure caused by nano PET. Thus, we treated the monolayer cell model exposed to nano PET with EcN, EcN_T_, EcN@L, EcN_T_@L, and PBS, followed by analyzing the permeability of the cell monolayer. Compared to other groups, the cell monolayer treated by EcN_T_ demonstrated minimal permeability as they showed lowest fluorescence in the sublayer, implying that EcN_T_ could repair intestinal damage caused by nano PET. Besides, exposure to nano PET significantly elevated proinflammatory cytokines in Caco‐2 cells, including tumor necrosis factor‐α (TNF‐α), inducible nitric oxide synthase (iNOS), and interleukin‐6 (IL‐6) (Figure 3c–e). Treatment with engineered probiotics, EcN_T_ and EcN_T_@L, effectively reduced the expression of these cytokines, suggesting their potential in alleviating inflammation. Importantly, these probiotics did not adversely affect Caco‐2 cell growth, indicating their safety for potential therapeutic applications (Figure S15, Supporting Information). The expressing level of TGF‐β in Caco‐2 cells treated with EcN_T_ and EcN_T_@L was three times higher than that of cells treated with EcN and EcN@L (Figure 3f). Therefore, EcN_T_ and EcN_T_@L showed efficiency in repairing gut barrier damages and modulating inflammatory reaction of intestinal cells upon nano PET treatment.

We hypothesized that the protective effect of probiotics against nanoplastics is due to the reduced cellular uptake of nano PET since both probiotics and TGF‐β were effective for restoration of cytomembrane, preventing the cell uptake of exogenous substances.^[^ ^48^, ^49^ ^]^ We treated Caco‐2 cells with various formulations including nano PET‐FITC, EcN, EcN@L, EcN_T_, and EcN_T_@L, followed by analysis through fluorescence microscopy and flow cytometry. The results displayed a pronounced green fluorescence in cells exposed to nano PET‐FITC, while a subsequent 4 h incubation with EcN significantly decreased the fluorescence intensity (Figure S16, Supporting Information), indicating that EcN effectively inhibited the cellular uptake of nano PET. Moreover, treatment with EcN_T_ and EcN_T_@L resulted in a 58.2% reduction in FITC^+^ cells, demonstrating a greater efficacy compared to EcN and EcN@L (Figure 3g; Figure S17, Supporting Information). These findings suggested that the secretion of TGF‐β played a critical role in diminishing nano PET uptake. Importantly, EcN_T_@L not only reduced the uptake of nano PET but also contributed to the restoration of cell membrane integrity and the inhibition of intracellular inflammation, highlighting its potential therapeutic relevance.

To better demonstrate the ability of EcN_T_@L on regulating inflammatory responses in gastrointestinal tract, we explored the effect of EcN_T_@L in the treatment of nano PET‐induced intestinal injury. Long time administration (28 d) of nano PET resulted in severe inflammation in the gastrointestinal tract (Figure S18, Supporting Information), and short time exposure (7 d) of nano PET induced slight cell damage and epithelial edema (Figure S19, Supporting Information). Meanwhile, oral administration of nano PET (2 mg mL^−1^) for 7 days resulted in gut barrier damage and increased the intestinal permeability (Figure S20, Supporting Information). Hence, we administrated the mice with nano PET continuously for 7 d to obtain a moderate inflammation model, and observed that EcN_T_@L ameliorated the inflammatory reaction of gastrointestinal tract (Figure S21, Supporting Information). Collectively, benefiting from the anti‐inflammatory activity of probiotics as well as the secretion of TGF‐β, EcN_T_@L suppressed nano PET‐associated intestinal inflammation.

### 2.4 EcN_T_@L Inhibited Nano PET‐Associated Gut Infections by Activating the NF‐κB Pathway

**Figure 4:**
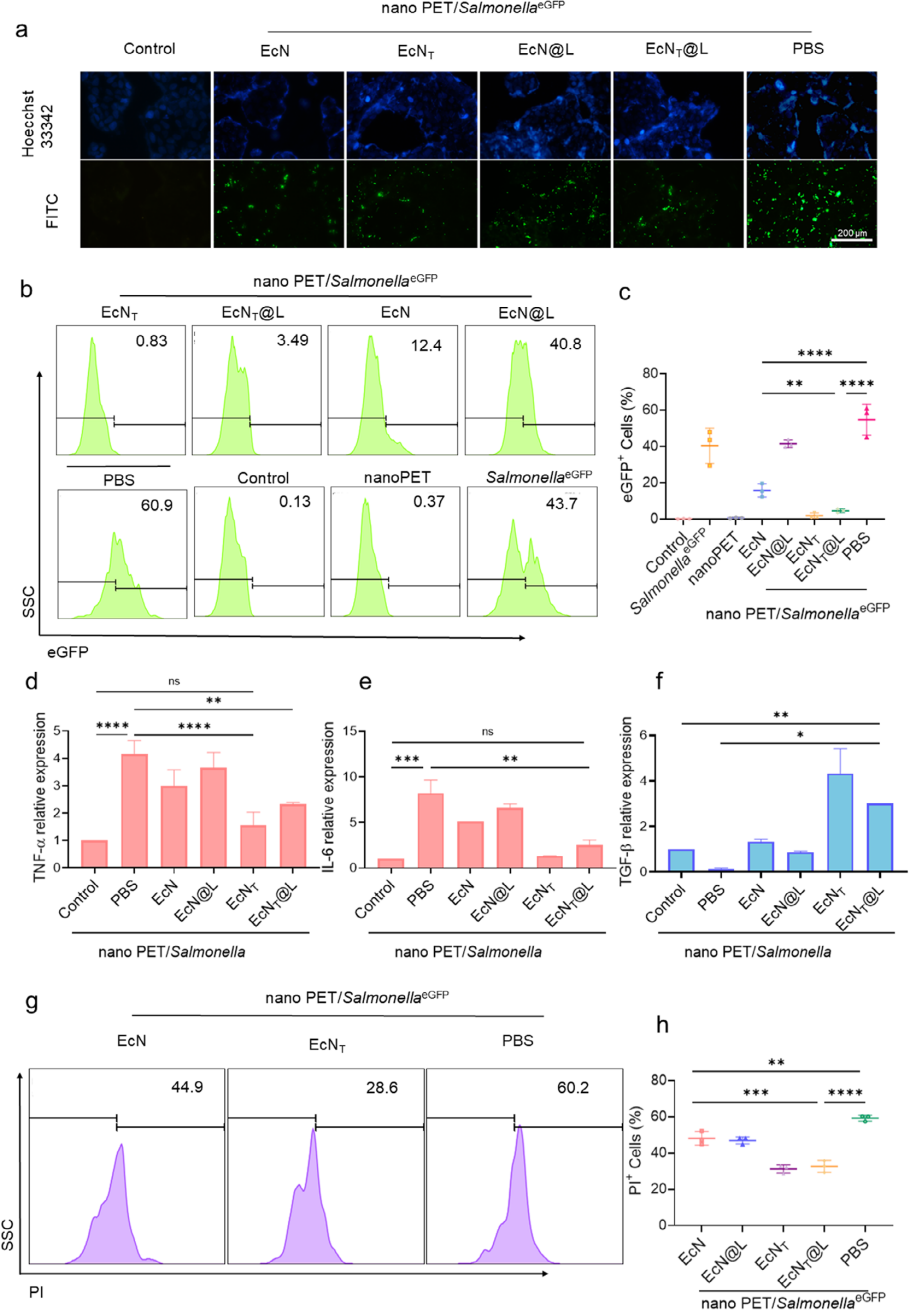
EcN_T_@L prevented nano PET‐associated *Salmonella* infections in vitro. a) Fluorescence images of Caco‐2 cells treated with PBS, EcN, EcN_T_, EcN@L, and EcN_T_@L post incubation with nano PET/*Salmonella* ^eGFP^. Scale bar: 200 mm. b,c) Flow cytometry assay (b) and the level of eGFP^+^ cells (c) in cells treated with PBS, EcN, EcN_T_, EcN@L, and EcN_T_@L post incubation with nano PET/*Salmonella*. d–f) Relative expression of TNF‐α (d), IL‐6 (e), and TGF‐β (f) in Caco‐2 cells treated with PBS, EcN, EcN_T_, EcN@L, and EcN_T_@L post incubation with nano PET/*Salmonella*. g,h) Flow cytometry assay (g) and the level of PI^+^ cells (h) in cells treated with PBS, EcN, EcN_T_, EcN@L, and EcN_T_@L post incubation with nano PET/*Salmonella*. *n* = 3, *p ≤ 0.05, **p ≤ 0.01, ***p ≤ 0.001, ****p ≤ 0.0001.

The interplay between compromised immune defenses and impaired barrier functions in the inflamed gastrointestinal tract significantly diminishes resistance to pathogenic invasions relative to healthy systems.^[^ ^50^, ^51^ ^]^ Recent findings indicate that the introduction of nano PET contributes to alterations in gut microbiota diversity, notably resulting in an increased abundance of various pathogenic species.^[^ ^52^ ^]^ We found that as the concentration of nano PET increased, the intracellular presence of *Salmonella* escalated as well (Figure S22, Supporting Information), suggesting that nano PET accelerated the cellular uptake of *Salmonella*. We incubated cells with nano PET/*Salmonella* ^eGFP^, followed by treatment with EcN, EcN@L, EcN_T_, and EcN_T_@L, and then observed the cells under fluorescence microscopy. EcN_T_ and EcN_T_@L demonstrated the highest inhibiting efficacy on cellular uptake of *Salmonella* ^eGFP^ among all treated groups, as less *Salmonella* ^eGFP^ appeared in cells treated with EcN_T_ and EcN_T_@L (**Figure** **4**a; Figure S23, Supporting Information). The level of eGFP^+^ cells decreased by 85.2% post treatment by EcN_T_ and EcN_T_@L (Figure 4b,c), which indicated that EcN_T_ and EcN_T_@L inhibited cellular uptake of *Salmonella*.

*Salmonella* infections pose significant health challenges by triggering severe inflammatory reactions that ultimately lead to cellular apoptosis and necrosis.^[^ ^53^ ^]^ We explored the efficacy of EcN_T_ and EcN_T_@L in modulating the inflammatory response associated with such infections. EcN_T_ and EcN_T_@L decreased the relative expression level of TNF‐α, IL‐6, and iNOS (Figure 4d,e; Figure S24, Supporting Information), and increased the level of IL‐10 and TGF‐β in cells treated by nano PET/*Salmonella* (Figure 4f; Figure S25, Supporting Information). These results underscored a superior anti‐inflammatory capability of EcN_T_ and EcN_T_@L compared to EcN and EcN@L. Live/dead staining assays revealed a marked reduction in PI^+^ cells following treatment with nano PET/*Salmonella*, indicating that EcN_T_ and EcN_T_@L effectively protected cellular viability against *Salmonella*‐induced cytotoxicity (Figure 4g,h). Therefore, EcN_T_ and EcN_T_@L not only mitigated inflammation but also inhibited cell death linked to nano PET/*Salmonella* exposure.

**Figure 5:**
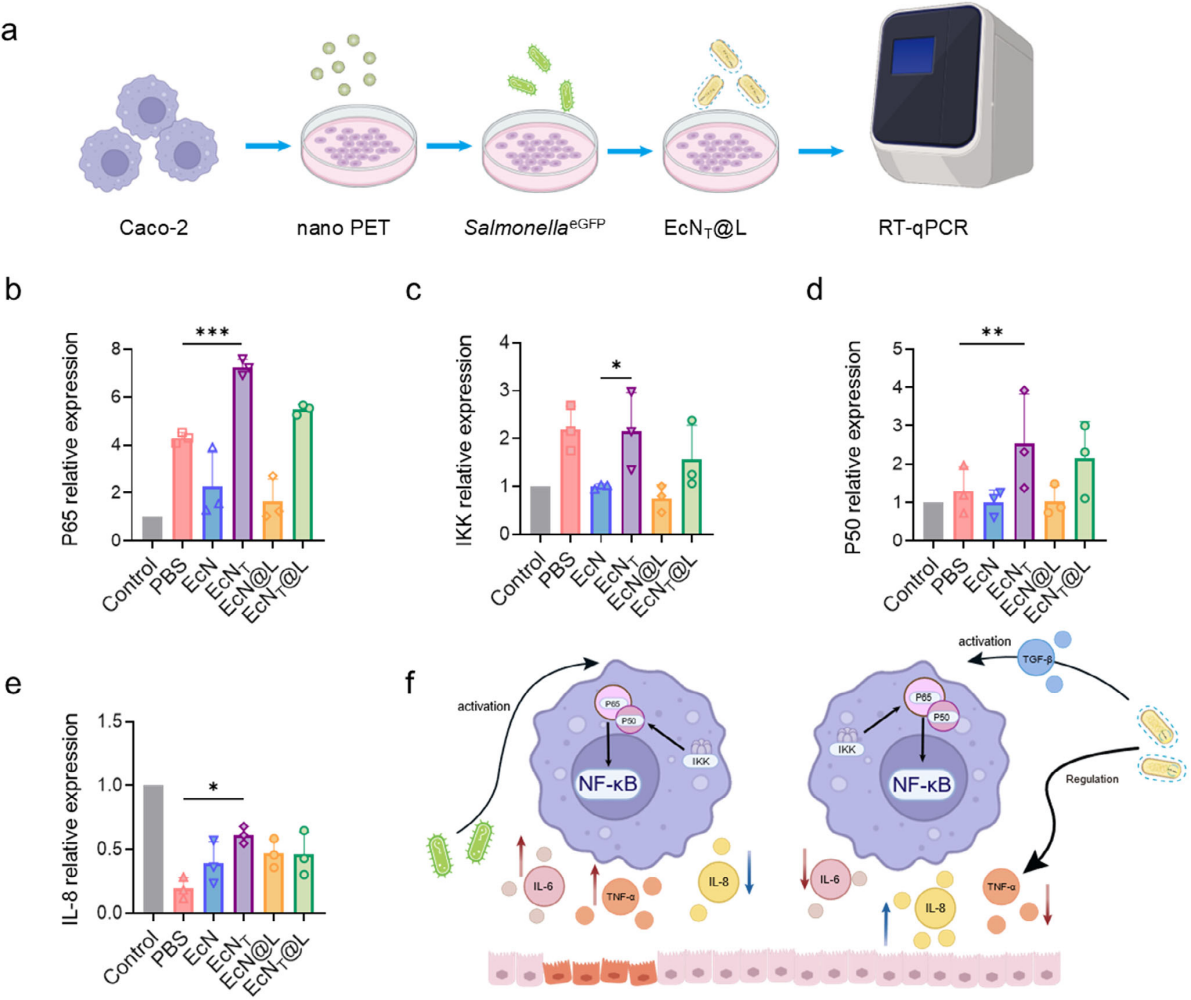
EcN_T_@L activated the NF‐κB pathway without inducing excess inflammation. a) Experimental design of RT‐qPCR for detecting main markers on NF‐κB pathway in cells. b–e) Relative expression of P65 (b), IKK (c), P50 (d), and IL‐8 (e) in Caco‐2 cells treated with PBS, EcN, EcN_T_, EcN@L, and EcN_T_@L. f) EcN_T_@L activates the NF‐κB pathway to eliminate *Salmonella* and suppressed excess inflammation. *n* = 3, *p ≤ 0.05, **p ≤ 0.01, ***p ≤ 0.001.

The activation of NF‐κB pathway was closely related to *Salmonella* infections.^[^ ^54^ ^]^ We investigated the anti‐infectious mechanism of EcN_T_@L in cells treated with nano PET/*Salmonella* by detecting key protein expression in NF‐κB pathway. Through RT‐qPCR analysis of key NF‐κB pathway proteins in Caco‐2 cells (**Figure** **5**a), we noted a significant upregulation of important NF‐κB subunits P65 and P50, as well as IKK, in response to EcN_T_ and EcN_T_@L, despite *Salmonella* treatment alone (the PBS group) had a certain effect on activating the NF‐κB pathway (Figure 5b–d). In contrast, EcN inhibited NF‐κB pathway activation since it could be used as an immunosuppressive agent.^[^ ^55^ ^]^ Therefore, EcN and EcN@L exhibited minimal effects on these markers. These results together indicated that TGF‐β secretion by EcN_T_ played a pivotal role in positively activating the NF‐κB pathway. Correspondingly, we observed a substantial increase in IL‐8 expression in cells treated with EcN_T_ and EcN_T_@L (Figure 5e), which could induce the migration and activation of immune cells and promote an immune response for pathogen elimination. Meanwhile, the treatment with these engineered probiotics led to the downregulation of proinflammatory cytokines IL‐6 and TNF‐α (Figure 4d,e), highlighting the ability of EcN to modulate inflammatory responses. Collectively, the engineered probiotics facilitated the activation of immune responses while alleviating cellular inflammation, all achieved via the NF‐κB pathway, preventing the occurrence of inflammatory storms.

### 2.5 EcN_T_@L Treated Nano PET‐Associated *Salmonella* Infections In Vivo

**Figure 6:**
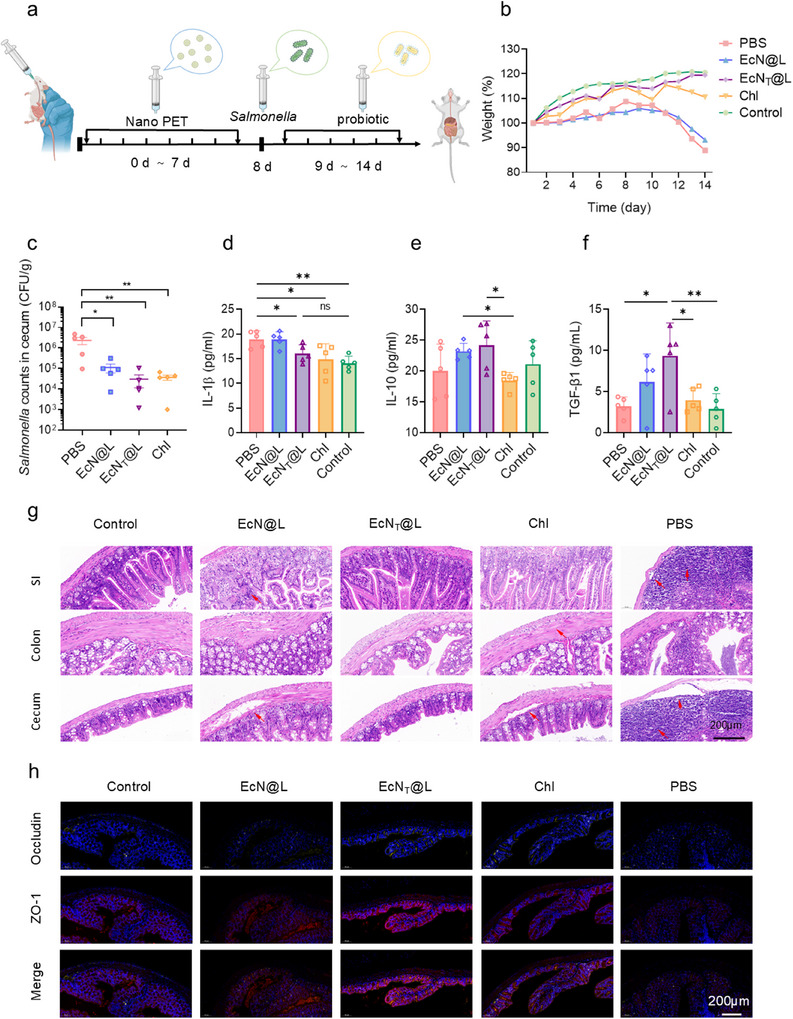
Therapeutic effects of EcN_T_@L on nano PET‐associated *Salmonella* infection. a) Schematic diagram of experimental protocol for treatment of nano PET‐associated *Salmonella* infection in mice. The mice were given nano PET (2 mg mL^−1^) from day 0 to day 6 by gavage, and then administrated with *Salmonella* (1 × 10^7^ CFU) on day 7. The infected mice were treated with PBS, Chl, EcN@L, and EcN_T_@L (1 × 10^8^ CFU) on day 8 for 5 continuous days. b) Bodyweight of mice during the experimental procedure. c) The counts of *Salmonella* in cecum of mice post treatment. d–f) The levels of IL‐1β (d), IL‐10 (e), and TGF‐β1 (f) serum of mice post treatment. g) HE staining of SIs, colons, and cecum in mice post treatment. Scale bar: 200 µm. h) Immunofluorescence staining of colon sections to visualize the expression patterns of ZO‐1 (red) and Occludin (yellow). Scale bar: 200 µm. *n* = 5, *p ≤ 0.05, **p ≤ 0.01.

To investigate the effect of EcN_T_@L on nano PET‐induced *Salmonella* infections in vivo, mice were administrated with a 7‐d oral gavage of nano PET, followed by *Salmonella* infection and different treatments comprising PBS, EcN@L, EcN_T_@L, and chloramphenicol (Chl) (**Figure** **6**a). The results demonstrated that, in comparison to the control groups treated with PBS, EcN@L, and Chl, the EcN_T_@L treatment significantly mitigated weight loss observed in the nano PET/*Salmonella* infected mice (Figure 6b). Notably, EcN_T_@L exhibited superior efficacy in eradicating *Salmonella* from major organs and the gastrointestinal tract, outperforming the EcN@L treatment and showing a comparable antibacterial effect to that of the commercially available Chl (Figure 6c; Figure S26, Supporting Information). Analysis of serum cytokine levels revealed that EcN_T_@L treatment resulted in a reduction of proinflammatory interleukin‐1 beta (IL‐1β) levels compared to the PBS group while concomitantly elevating the concentrations of the anti‐inflammatory cytokines IL‐10 and transforming growth factor‐beta 1 (TGF‐β1) compared to the Chl group (Figure 6d–f), indicative of its potential to alleviate gut inflammation and combat infection. Histological evaluations through hematoxylin–eosin (HE) staining illustrated a pronounced prevalence of severe epidermal edema, inflammation, and infiltration of inflammatory cells within the liver, spleen, and kidneys of mice subjected to PBS and Chl treatment (Figure S27, Supporting Information). In contrast, both the PBS and EcN@L groups exhibited notable inflammation and mucosal swelling in the gastrointestinal tract, whereas tissues from the EcN_T_@L‐treated group displayed negligible inflammatory responses (Figure 6g). Compared to the PBS group, EcN_T_@L allowed the amelioration of the permeability of gut barriers (Figure S28, Supporting Information) and the upregulation of ZO‐1 and Occludin in colons (Figure 6h), indicating that EcN_T_@L improved the tight junction and barriers of colons that were damaged by nano PET/*Salmonella*. These findings underscored the dual role of EcN_T_@L in diminishing the secretion of proinflammatory mediators and facilitating the release of anti‐inflammatory cytokines, thereby playing a crucial role in modulating the inflammatory response and promoting the repair of intestinal barrier integrity.

### 2.6 EcN_T_@L Regulated the Immune Microenvironment and Microbiota in Gut

**Figure 7:**
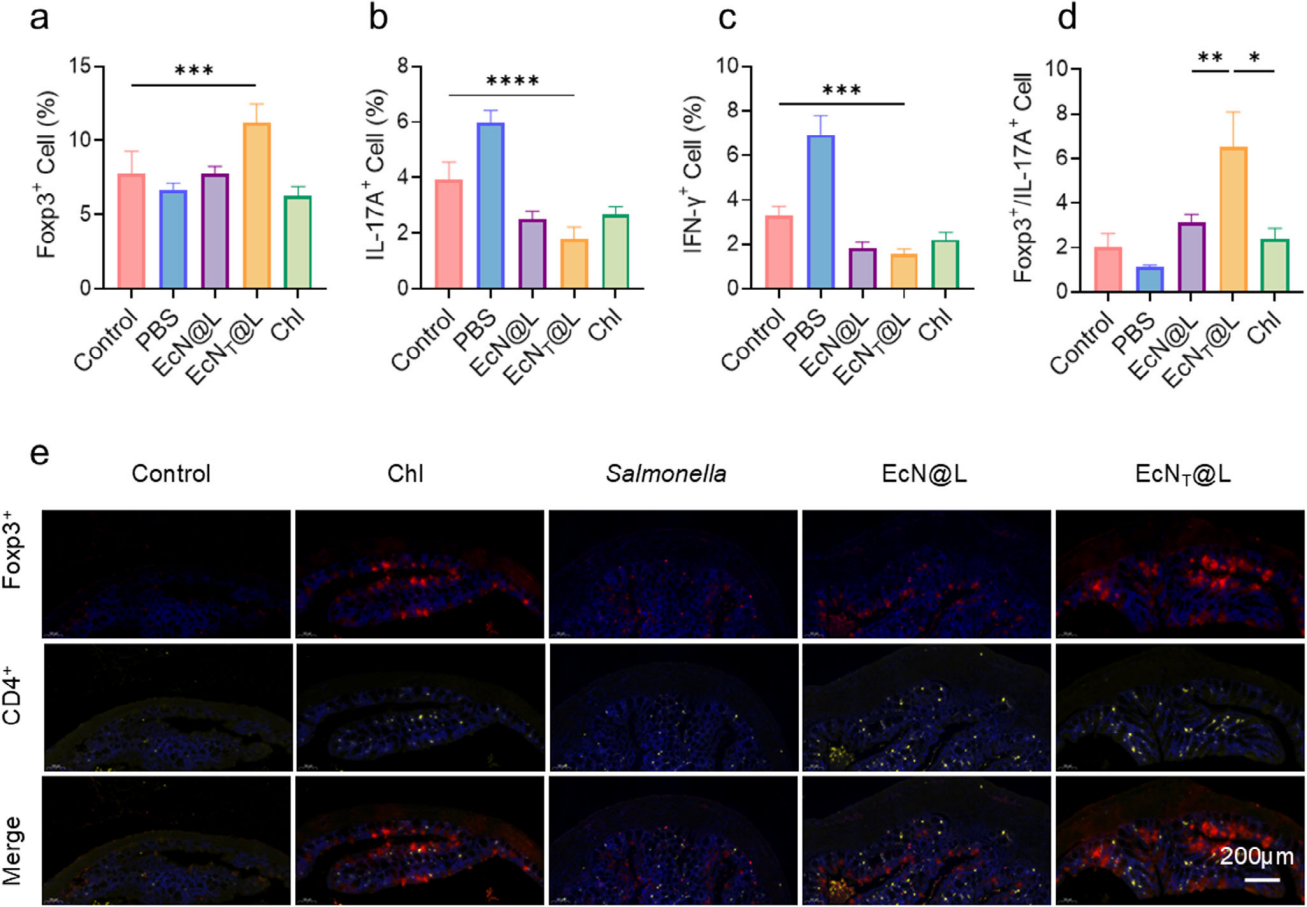
EcN_T_@L regulated the balance of Treg and Th cells. a–c) The level of CD4^+^Foxp3^+^ (a), CD4^+^IL‐17A^+^ (b), and CD4^+^IFN‐γ^+^ (c) cells in mesenteric lymph nodes of mice post treatment. d) The ratio between CD4^+^Foxp3^+^ and CD4^+^IL‐17A^+^ cells. e) Immunofluorescence staining of colon sections to visualize the expression patterns of Foxp3 (red) and CD4 (yellow). The nuclei were stained with DAPI (blue). Scale bar: 200 µm. *n* = 5, *p ≤ 0.05, **p ≤ 0.01, ***p ≤ 0.001, ****p ≤ 0.0001.

The balance of T regular cells and T helper cells is relevance to the inflammatory reaction in mice models, relating to their recovery of gastrointestinal tract.^[^ ^56^ ^]^ Recent studies have demonstrated that nanoplastics modulate the intestinal ecosystem through direct interactions with macrophages.^[^ ^57^, ^58^ ^]^ Activation of the NF‐κB pathway and administration of EcN have been reported to promote immune T cell response.^[^ ^59^ ^]^ We extracted mouse mesenteric lymph node cells to detect the effect of EcN_T_@L on T regular cells (Treg) and T helper cells (Th). TGF‐β was essential for the differentiation of Treg cells from CD4^+^ cells, and Treg cells are closely related to the secretion of a series of anti‐inflammatory factors, playing an essential role in maintaining immune tolerance and immune homeostasis.^[^ ^60^ ^]^ Highest amounts of Foxp3^+^ cells were found in EcN_T_@L groups among all treated groups (**Figure** **7**a), which illustrated that EcN_T_@L remarkably increased the level of Treg cells in the colon of mice. Besides, EcN@L, EcN_T_@L, and Chl decreased the level of IL‐17A^+^ and IFN‐γ^+^ cells (Figure 7b,c). Compared with EcN@L and Chl, mice treated with EcN_T_@L showed higher ratio between Foxp3^+^ and IL17A^+^ cells (Figure 7d), suggesting that EcN_T_@L was more efficient in restoring the balance between anti‐inflammatory and proinflammatory cells. Furthermore, highest red fluorescence indicated Foxp3 appeared in colons of mice treated by EcN_T_@L (Figure 7e), owing to the ability of TGF‐β to improve Treg differentiation. Therefore, EcN_T_@L promoted the recovery of infectious gastrointestinal tract by modulating the balance between proinflammatory and anti‐inflammatory CD4^+^ T cells.

**Figure 8:**
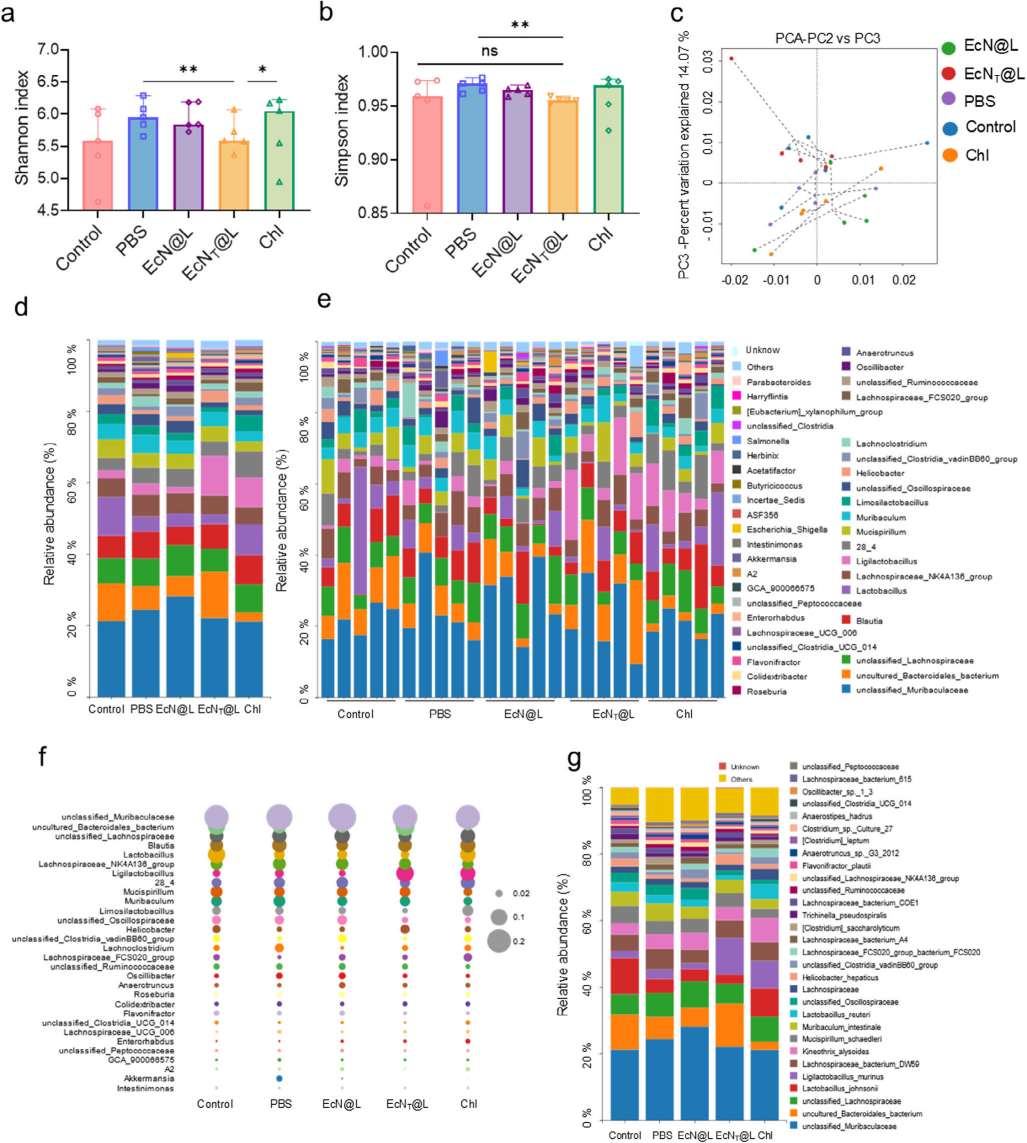
EcN_T_@L modulated gut microbiota of nano PET/*Salmonella* infected mice. a,b) Shannon (a) and Simpson (b) index of gut microbiota in mice post treatment. c) PCA analysis of gut microbiota in mice post treatment. d,e) The relative abundance of genus in gut microbiota of mice post treatment. f) Bubble chart of genus composition in gut microbiota of mice post treatments. g) Relative abundance of phylum in gut microbiota of mice post treatment. *n* = 5, *p ≤ 0.05, **p ≤ 0.01.

The gut microbiota plays a pivotal role in maintaining the integrity of gut barriers and defending against pathogenic invaders.^[^ ^61^ ^]^ However, gut infection frequently induced the dysbiosis of gut microbiota.^[^ ^62^ ^]^ Given to the ability of EcN on modulating gut microbiota,^[^ ^63^ ^]^ we suspected that EcN_T_@L repaired gut barrier by restoring gut microbiota. Our findings demonstrated that *Salmonella* infection significantly altered gut microbiota diversity, as evidenced by increased Shannon and Simpson indexes in the PBS group (**Figure** **8**a,b). In contrast, EcN_T_@L exhibited superior efficacy in restoring microbial diversity compared to EcN@L and Chl, evidenced by clustering patterns observed through principal component analysis (PCA). The samples in PBS, EcN@L, and Chl gathered in another cluster (Figure 8c). *Salmonella* competed with obligate anaerobes in the gut, resulting in an increase of strict anaerobes such as *Blautia* (Figure S29a, Supporting Information), and increased the abundance of inflammatory *Proteobacteria* (Figure 8d; Figure S29b, Supporting Information). Besides, high level of *Salmonella* appeared post infections (Figure S29c, Supporting Information). Among the treated groups, EcN_T_@L effectively reduced the abundance of *Salmonella* and increased the abundance of intestinal probiotics such as *Ligilactobacillus* (Figure 8f; Figure S29d, Supporting Information), indicating that EcN_T_@L improved the gut mucosa and metabolic functions. Moreover, EcN_T_@L increased the abundance of *Ligilactobacillus_murinus*, demonstrating that EcN_T_@L enhanced intestinal nutrition absorption and energy extraction (Figure 8g). These results indicate that EcN_T_@L not only enhances gut mucosal health and metabolic functions but also fosters a favorable microbial balance critical for intestinal homeostasis. Overall, EcN_T_@L allowed the restoration of gut microbiota, modulated immune responses, and mitigated inflammatory damage within the gastrointestinal tract stimulated by PET‐associated *Salmonella* infections.

## 3 Discussion

**Figure 9:**
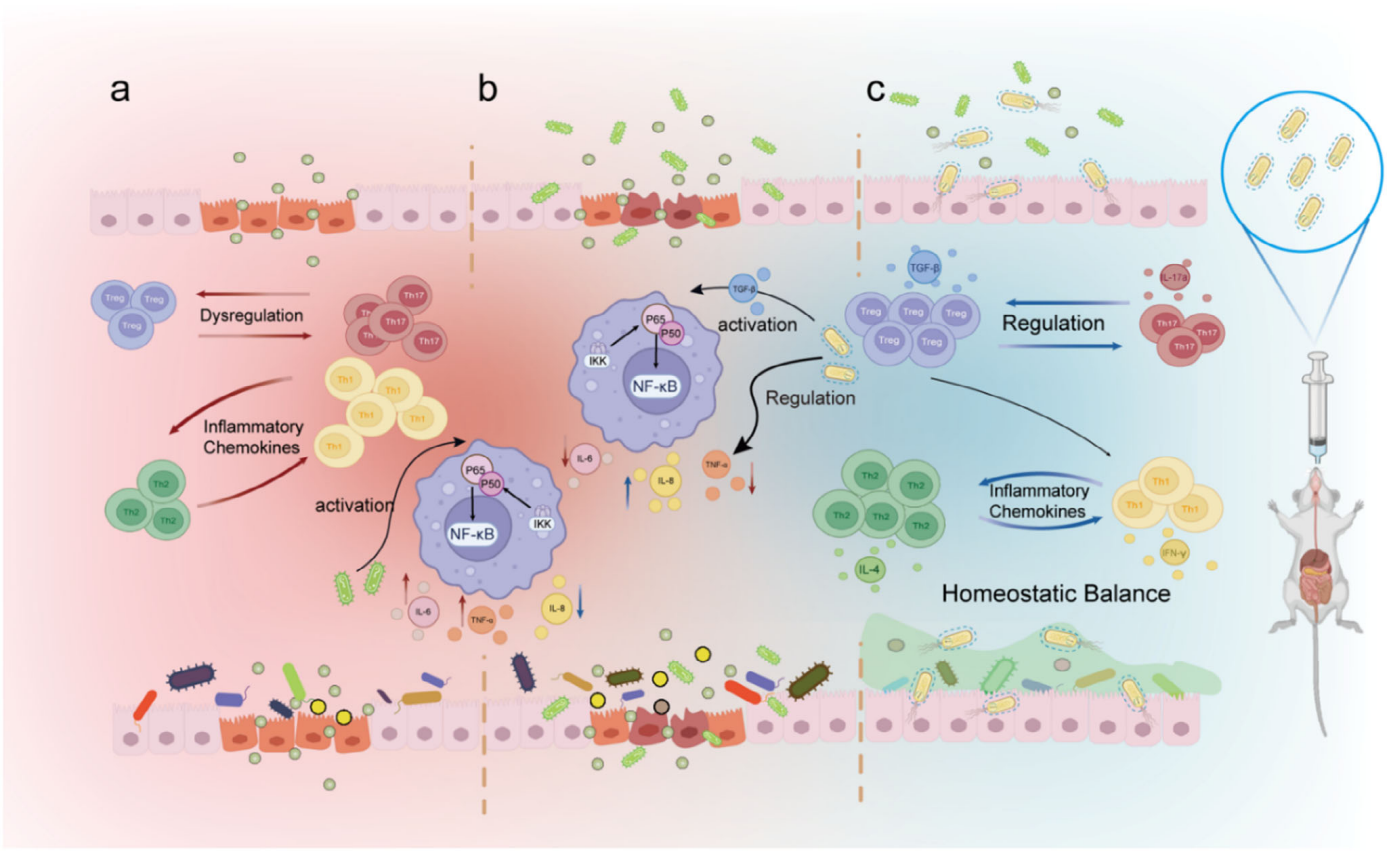
Engineered probiotics for the therapy of PET‐associated gut barrier dysfunction. EcN was transferred with a plasmid encoding TGF‐β and then capsuled with L100‐55 to obtain EcN_T_@L. L100‐55 improves the bioaviability and retention of EcN_T_ in gastrointestinal tract, and thus activating NF‐κB pathway and relieving nano PET‐associated inflammation and infections.

Micro‐ and nanoplastics pose a significant threat to human gastrointestinal health once they enter the food chain. Exposure to nano PET has been linked to various complications, including intestinal mucosal inflammation, pathogenic bacterial invasion, immune imbalances, and disruptions in the gut microbiota (**Figure** **9**a). Despite growing concerns about gut disorders caused by micro‐ and nanoplastics, clinical drugs are still under development, with limited results in clinical treatment. Several agents have been developed in the lab to alleviate gut inflammation caused by nanoplastics, such as the polyphenolic epigallocatechin‐3‐gallate, which has been shown to mitigate polystyrene‐induced intestinal inflammation.^[^ ^64^ ^]^ Additionally, strategies aimed at enhancing intestinal motility or degrading microplastics have been developed, which help alleviate microplastic‐induced gut damage.^[^ ^65^, ^66^ ^]^ However, these treatments show limited efficacy and struggle to provide comprehensive repair of intestinal injuries. In contrast, the focus of our research, EcN_T_@L, not only modulates the immune system to reduce intestinal inflammation but also restores the homeostasis of the gut microbiota, offering a more holistic approach to repairing microplastic‐induced intestinal damage. In the presence of *Salmonella* infection (Figure 9b), the naturally activated NF‐κB signaling pathway triggers excessive immune responses, characterized by elevated levels of proinflammatory cytokines. Our engineered EcN_T_@L strain effectively regulates the NF‐κB pathway by modulating immune responses to target and eliminate pathogens, while simultaneously reducing inflammation (Figure 9c). The TGF‐β secreted by EcN_T_ helps restore balance between proinflammatory and anti‐inflammatory CD4^+^ T cells, promoting recovery in the affected gastrointestinal tract. Notably, the introduction of these probiotics resulted in significant shifts in the composition of the intestinal microbiota, increasing beneficial bacteria and decreasing pathogenic strains, ultimately helping to restore a healthy gut microbiome balance. Together, we pioneer the therapeutic use of engineered probiotics in alleviating nanoplastics‐associated gut disorders, potentially opening new avenues for controlling the risks posed by micro‐ and nanoplastics.

## 4 Conclusion

In summary, we have developed a doubly engineered probiotic therapeutic, EcN_T_@L, to treat nano PET‐associated gut inflammation and infections. Our strategy allows controlled, on‐demand release of both the probiotic cells and TGF‐β in the gut, improving their oral bioavailability and retention within the gastrointestinal tract. The TGF‐β secretion enables EcN_T_@L to effectively alleviate inflammation related to nano PET exposure by repairing cellular membranes. EcN_T_@L is effective against nano PET‐associated infections, both in vitro and in vivo, as demonstrated by its ability to activate the NF‐κB pathway in Caco‐2 cells while reducing inflammation. EcN_T_@L mitigates nano PET‐associated *Salmonella* infection by correcting the dysregulated CD4^+^ T cell response and restoring the gut microbiota. This study highlights the therapeutic potential of bioengineered probiotics for addressing nano PET‐related gut barrier dysfunction, paving the way for future diversification in probiotic therapies aimed at counteracting the adverse effects of environmental pollutants. Future research will focus on exploring the selectivity of probiotics toward different types of plastics or plastics with varying length scales, with the aim of developing universally applicable probiotic‐based therapeutics.

## 5 Experimental Section

### Materials

Eudragit L100‐55 was a gift from Evonik. Premix, FITC‐conjugated anti‐CD3, APC/Cyanine7‐conjugated anti‐CD4, APC‐conjugated anti‐IL‐4, PE‐conjugated anti‐Foxp3, Brilliant Violet 421‐conjugated anti‐IFN‐γ, and Brilliant Violet 605‐conjugated anti‐IL‐17A were obtained from Biolegend. Primers of pBBR1MCS2, pBV220, were synthesized by Sangon Biotech. FITC and CY5 were from Solarbio. Total RNA extracted kit and cDNA kit were bought from Sangon Biotech. ELISA kits of IL‐10, TGF‐β1, IL‐1β, and IL‐4 were obtained from Ruixin Biotech. All other regents were used as received without further purification. *Escherichia coli* Nissle1917 (EcN) was cultured in LB medium at 37 °C. pBBR‐mCherry was purchased from Biofilm. mCherry expressing EcN (EcN_m_) was constructed by thermal shock conversion pBBR‐mCherry in to receptive EcN. TGF‐β (cf. accession No. X52498) was synthesized by Sangon Biotech, and then inserted into pBBR plasmid by homologous recombination. *Salmonella* marked with eGFP (*Salmonella*‐eGFP) was constructed by transferring pBBR‐eGFP into wild type *Salmonella* via electrical conversion method.

### Preparation and Characterization of Nano PET and Nano PET‐FITC

To prepare nano PET, bulk PET was dissolved with hexafluoroisopropanol (HFIP), and then added into deionized water at room temperature, resulting in precipitation of nano PET. The entire contents of the precipitation vessel were rotary evaporated under vacuum at 55 °C to remove residual HFIP. FITC‐conjugated nano PET (PET‐FITC) was obtained by adding FITC into the HFIP when prepared nano PET. The ζ‐potential and size distribution of nano PET and nano PET‐FITC were analyzed using Malvern Zetasizer nano ZS. The morphology of nano PET and nano PET‐FITC were observed under transmission electron microscope (TEM, Talos F200X G2).

### Preparation of EcN_T_@L

EcN‐TGF‐β (EcN_T_) with anti‐kanamycin ability was prepared through heat shock of pBBR‐TGF‐β plasmid to EcN receptive state. EcN_T_ was cultured in LB medium containing kanamycin (50 mg mL^−1^) for 4 h before surface modification, and then the bacteria were collected by centrifugation at 4000 × *g*. EcN_T_ was suspended in 1 mL of cold CaCl_2_ solution (12.5 mm), and shaken at room temperature for 5 min. 100 µL of L100‐55 (5 mg mL^−1^) and 10 µL of HCl (10%) was added into the dispersion sequentially, and then treated with vortex for 10 s. EcN_T_@L was obtained post washing with PBS and centrifugation, and stored at 4 °C before use. Living bacteria in EcN_T_ and EcN_T_@L were counted by plate counting method. Besides, 1 × 10^5^ CFU of EcN_T_ and EcN_T_@L was added to a 96‐well plate, respectively, and their absorbance at 600 nm was recorded with a microplate reader every 30 min.

### Characterization of ECN_T_@L

EcN_T_ was cultured in 4 mL LB at 37 °C for 12 h at 150 rpm, and then centrifuged at 6000 rpm for 5 min. Subsequently, 100 µL of the supernatant was taken and it was detected in accordance with the instructions of the ELISA kit. To characterize the morphology of EcN_T_ and EcN_T_@L, 10 µL of bacteria (1 × 10^8^ CFU mL^−1^) was dispersed in 1× PBS and deposited on 300‐mesh grids with carbon membrane. The samples were washed with ultrapure water after 15 min incubation, and then they were observed under TEM (Talos F200X G2) after drying at room temperature. To better display their surface characteristic, EcN_T_ and EcN_T_@L were observed by using scanning electron microscope (SEM, Thermo). The bacteria were fixed in 2.5% glutaraldehyde solution for 2 h at room temperature and serially dehydrated in 30%, 50%, 70%, 80%, 90%, and 100% ethanol for 15 min. Besides, the average size and ζ‐potential of EcN_T_ and EcN_T_@L were determined by Malvern Zetasizer nano ZS. To assay the encapsulating efficiency of L100‐55, Cy5 was conjugated on L100‐55 through dehydration synthesis, meanwhile, EcN_m_ was used to form EcN_m_@Lc. Then EcN_m_ and EcN_m_@Lc were analyzed with flow cytometry (Beckman CytoFlex, USA). 1 × 10^8^ CFU of EcN_T_ and EcN_T_@L was dried at 55 °C, and then mixed with KBr at a ratio of 1:100 for Fourier transform infrared spectrometer (Frontier, Perkin Elmer).

### Sodium Dodecyl Sulfate‐Polyacrylamide Gel Electrophoresis (SDS‐PAGE)

To assess the expression of TGF‐β, EcN, EcN_T_, and EcN_T_@L were cultured in LB broth for 2 h, and then supplemented with Kan (100 µg mL^−1^) for another 12 h. The bacteria were collected and centrifuged to separate the bacteria pellets for SDS‐PAGE analysis. 1 × 10^8^ CFU of EcN, EcN_T_, and EcN_T_@L was washed with PBS for three times, and then resuspended with 60 µL of SDS‐PAGE loading buffer and boiled for 30 min. The above protein samples were separated in 15% SDS‐PAGE gel, and stained with 0.25% coomassie brilliant blue glue for 1 h. The samples were observed and recorded by using BioRed imaging after decolorization.

### Survival Rate of ECN_T_@L in Simulated Fluids

In order to investigate the on‐demand performance of L100‐55 coating on bacteria in vitro, cell viability of EcN_T_ and EcN_T_@L was evaluated in SGF and SIF. To obtain SGF, 3.84 mL of hydrochloric acid was diluted with 1000 mL of ultrapure water, and then pepsin (10 g) was dissolved with the former solution. 1 × 10^9^ CFU of EcN_T_ and EcN_T_@L was added in SGF, and certain volume of bacteria was taken at 0, 0.5, 1.0, and 2.0 h. To obtain SIF, 6.8 g of KH_2_PO_4_ was dissolved with 250 mL of ultrapure water, and the proper volume of NaOH and trypsin (10 g) was added into KH_2_PO_4_ solution until pH was 7.2. 1 × 10^5^ CFU of EcN_T_ and EcN_T_@L was added in SGF, and samples were collected at 0, 1.0, 2.0, 4.0, and 6.0 h post incubation. The amounts of living bacteria were counted by spreading bacterial dispersion on kanamycin containing LB agar plates.

### Cell Viability of Nano PET and EcN_T_@L

Caco‐2 cells were cultured with DMEM supplied with 10% FBS and 1% penicillin–streptomycin in a 5% CO_2_ incubator at 37 °C. 1 × 10^4^ cells were added in a 96‐well plate and cultured overnight. Then 100 µL of nano PET dispersion (2 mg mL^−1^), EcN, EcN@L, EcN_T_, and EcN_T_@L (10^8^ CFU mL^−1^) were added into the cells, respectively, and cultured for another 24 h. 10% CCK8 was added to the plates after washing with PBS, and incubated at 37 °C for 45 min. OD_450_ of the plates was measured by using a microplate reader.

Caco‐2 cells were cultured with DMEM supplied with 10% FBS and 1% penicillin–streptomycin in a 5% CO_2_ incubator at 37 °C. 1 × 10^4^ cells were added in a 96‐well plate and cultured overnight. Then 100 µL of nano PET dispersion (0, 1, 2, and 4 mg mL^−1^) was added into the cells, respectively, and cultured for another 24 h. 10% CCK8 was added to the plates after washing with PBS, and incubated at 37 °C for 45 min. OD_450_ of the plates was measured by using a microplate reader.

Raw264.7 cells were cultured with DMEM supplied with 10% FBS and 1% penicillin–streptomycin in a 5% CO_2_ incubator at 37 °C. 1 × 10^4^ cells were added in a 96‐well plate and cultured overnight. Then 100 µL of nano PET dispersion (0, 1, 2, and 4 mg mL^−1^) was added into the cells, respectively, and cultured for another 24 h. 10% CCK8 was added to the plates after washing with PBS, and incubated at 37 °C for 45 min. OD_450_ of the plates was measured by using a microplate reader.

### Effect of EcN_T_@L on Monolayer Cell Model

Caco‐2 cells were cultured in complete DMEM medium supplied with 10% FBS and 1% PS at 37 °C in a CO_2_ incubator. The monolayer cell model was established by seeding Caco‐2 cells into the uplayer of the transwell plate (0.4 µm pore size, 1 × 10^8^ cm^−2^ pore density, 8.4 mm diameter) at a concentration of 1 × 10^5^ cells per well. Media were changed every other day and cells were cultured for 9–11 days. Before the penetration studies, the barrier function was evaluated by measuring the permeability of FITC‐dextran. The inserts were transferred into another 24‐well plate supplied with 1 mL of DMEM medium, and then 200 µL of nano PET‐FITC (2 mg mL^−1^) was added into the insert with three parallels and incubated for 2 h. The basal media were collected and analyzed by using a fluorescence spectrophotometer. Afterward, PBS, EcN, EcN_T_, EcN@L, and EcN_T_@L (1 × 10^8^ CFU) were added into the inserts after they were washed with PBS for three times, and then incubated for another 4 h. Then 200 µL of FITC‐dextran (1 mg mL^−1^) was added into the inserts, and cultured for 3 h, and the basal liquid was collected and analyzed by using a fluorescence spectrophotometer.

### Cell Uptake of *Salmonella*, Nano PET, and EcN_T_@L

To study the cell uptake of nano PET in Caco‐2 cells, 1 × 10^6^ cells were incubated in a 24‐well plate, and cultured for 24 h. Then 0.5 mL of nano PET‐FITC (2 mg mL^−1^) was added into the plate, and cells were observed under fluorescence microscopy post incubation for 0.5, 1.0, 2.0, and 4.0 h.

To assess the cell uptake of nano PET in Caco‐2 cells post treatment of EcN_T_, 1 × 10^6^ cells were incubated in a 24‐well plate, and cultured for 24 h. Then 0.5 mL of nano PET‐FITC (2 mg mL^−1^) was incubated with cells for 2.0 h, and then 1 × 10^8^ CFU of EcN_T_ was added after washing with PBS. Cells were washed and observed under fluorescence microscopy at 0.5, 1.0, 2.0, and 4.0 h post incubation. To further display the efficacy of EcN_T_@L, 1 × 10^6^ cells were incubated in a 24‐well plate, and cultured for 24 h. Then 0.5 mL of nano PET‐FITC (2 mg mL^−1^) was incubated with cells for 2.0 h, and 1 × 10^8^ CFU of EcN, EcN_T_, EcN@L, and EcN_T_@L was added into the cells after removing nano PET‐FITC. Cells were harvested at 4 h post incubation, and then analyzed by flow cytometry. 10 000 cells were gated for further analysis.

To study cell uptake of *Salmonella* in Caco‐2 cells post incubation with nano PET and EcN_T_, 1 × 10^6^ cells were incubated in a 24‐well plate, and cultured for 24 h. Then 0.5 mL of nano PET (2 mg mL^−1^) was incubated with cells for 2.0 h. 1 × 10^6^ CFU of *Salmonella* ^eGFP^ was added into the cells after removing nano PET, and incubated for 2.0 h. Then 1 × 10^8^ CFU of EcN, EcN_T_, EcN@L, and EcN_T_@L was added into the cells, and incubated for another 4.0 h. Cells were washed and harvested after washing with PBS, and then analyzed by flow cytometry. 10 000 cells were gated for further analysis.

### Effect of EcN_T_@L on Cellular Inflammation

10^7^ Caco‐2 suspension was added to 12‐well plate. The culture plates were precultured in an incubator for 24 h (at 37 °C, 5% CO_2_). 500 µL of 2 mg mL^−1^ nano PET was added to the culture plate and incubated for 4 h in the incubator. Finally, the old medium was removed and the cells were washed twice with PBS. Total RNA Extractor (Trizol) kit was used to extract cell RNA. The RNA was reverse‐transcribed into cDNA using the HiScript IIQ RT SuperMix for qPCR kit and placed in the real‐time fluorescent quantitative PCR instrument according to the instructions.

### Effect of EcN_T_@L on Cell Infections

10^7^ Caco‐2 suspension was added to 12‐well plate. The culture plates were precultured in an incubator for 24 h (at 37 °C, 5% CO_2_). Then 0.5 mL of nano PET (2 mg mL^−1^) was incubated with cells for 2.0 h. 1 × 10^6^ of *Salmonella* was added into the cells after removing nano PET, and incubated for 2.0 h. Then 1 × 10^8^ CFU of EcN, EcN_T_, EcN@L, and EcN_T_@L was added into the cells, and incubated for another 4.0 h. Cells were washed and harvested after washing with PBS. Total RNA Extractor (Trizol) kit was used to extract cell RNA. The RNA was reverse‐transcribed into cDNA using the HiScript IIQ RT SuperMix for qPCR kit and placed in the real‐time fluorescent quantitative PCR instrument according to the instructions.

### Animals

Balb/c mice (female, 6–8 weeks) were from Hunan Slake Jingda Laboratory Animal Co. LTD. All animal experiments conducted in accordance with applicable laws were approved by the Institutional Animal Care and Use Committee of Hainan University (HNUAUCC‐2024‐00253).

### Effect of Nano PET on *Salmonella* Infections in an In Vitro Model

The small intestines were harvested and randomly divided into uniform length and washed with PBS for three times. Then the intestines were immersed in PBS containing nano PET (0, 0.5, 1.0, 1.5, and 2.0 mg mL^−1^), and incubated at 37 °C for 2.0 h. Afterward, 1 × 10^6^ CFU *Salmonella* were added into the intestines sequentially with three parallels, and incubated for another 4 h. PBS was used as a control. After washing with PBS for three times, the tissues were ground and diluted with PBS. The amount of *Salmonella* was counted by spreading the dispersion on streptomycin containing LB agar plate.

### Retention of EcN_T_@L Post Oral Administration

Mice were randomly divided into four groups (*n* = 3), and administrated with 0.2 mL of EcN, EcN_T_, EcN@L, and EcN_T_@L (1 × 10^9^ CFU) by gavage, respectively. Then their feces were collected and resuspended with 1 mL of PBS at 1, 2, 4, 6, 8, 12, and 24 h post administration. The amounts of EcN in feces were counted by spreading the samples on kanamycin containing LB agar plates.

### Biodistribution of EcN_T_@L Post Oral Administration

Mice were randomly divided into three groups (*n* = 3), and then 0.2 mL of PBS, EcN_m_, and EcN_m_@L (1 × 10^9^ CFU) was oral administrated. Then the fluorescence images of mice were recorded by IVIS (PerkinElmer, Lumina III) at 1, 2, 4, 6, and 24 h post gavage. Intestines were harvested and imaged by IVIS, and then ground for bacterial counting. The images were analyzed by Living Image 4.2, and the result was expressed in photon/second square centimeter/sphericity (p/s/cm^2^/sr).

### Effect of Nano PET on Healthy Mice

Mice were divided into four groups (*n* = 5) and they were oral administrated with 0.2 mL of nano PET (0.1, 1.0, and 2.0 mg mL^−1^) every day. PBS‐administrated mice were used as controls. Mice were sacrificed post a 7‐day administration and a 28‐day administration, and intestines were collected for HE staining. The mice orally administrated with PBS, 1 mg mL^−1^, and 2 mg mL^−1^ nano PET for 7 days were fasted for 24 h, and 100 µL of FITC‐dextran (4 kDa, 400 mg kg^−1^ bodyweight) was administrated by gavage. Blood samples were collected and centrifuged at 4 h post administration, and the samples were determined by a microplate fluorometer with excitation at 490 nm and emission at 530 nm.

### Treatment of EcN_T_@L on *Salmonella* Infectious Models

Mice were randomly divided into five groups (*n* = 5), and then four groups of them were administrated with 0.1 mL of nano PET (2 mg mL^−1^) every day. The mice were administrated with 0.2 mL of *Salmonella* (1 × 10^7^ CFU) via oral gavage post a 7‐day administration. On the other day, mice were treated with 0.2 mL of PBS, EcN@L (1 × 10^9^ CFU mL^−1^),^[^ ^36^, ^41^ ^]^ EcN_T_@L (1 × 10^9^ CFU mL^−1^), and chloramphenicol (100 µg mL^−1^) through oral administration for five continuous days. Mice without treatment were used as controls. Bodyweight of mice was recorded ever day during the experiment. All mice were sacrificed at the sixth day post treatment. Before sacrifice, the mice were fasted for 12 h, and 100 µL of FITC‐dextran (4 kDa, 400 mg kg^−1^ bodyweight) was administrated by gavage. Blood samples were collected and centrifuged at 4 h post administration, and the samples were determined by a microplate fluorometer with excitation at 490 nm and emission at 530 nm, and the gastrointestinal tracts and main organs were harvested and collected. Parts of the livers, spleens, kidneys, small intestines, cecum, and colons were homogenized in PBS for *Salmonella* counting. Half of these organs were gathered for histopathological assessment through HE staining. Besides, blood was obtained from mice, and the concentrations of IL‐1β, IL‐4, IL‐10, and TGF‐β in serum were determined by ELISA kits. Furthermore, the colons were further fixed and stained with fluorescence IF555‐Tyramide‐Foxp3, IF647‐Tyramide‐CD4, IF647‐Tyramide‐ZO‐1, IF555‐Tyramide‐Occludin antibody, and then scanned under fluorescence microscopy.

The spleen and mesenteric lymph nodes were collected and ground with syringe, and the cells were collected by centrifugation at 2000 rpm after filtering with 40 µm cell screen. The cells were resuspended in PBS and stained with APC‐CD4 and FITC‐CD3, and incubated for 45 min at room temperature. The cells were immobilized in 4% paraformaldehyde at room temperature for 30 min after washing with PBS for three times. After washing with PBS, the cells were incubated with PE‐Foxp3, APC‐IL‐4, and Brilliant Violet 605‐IL‐17A and Brilliant Violet 421‐IFN‐γ at room temperature for 45 min. The stained cells were analyzed by using the flow cytometer assay after washing with PBS.

### Gut Microbiota Analysis

The harvested cecum were quick‐frozen in liquid nitrogen for 16s RNA sequencing analysis. Library construction and sequencing include: after extracting the total DNA of the sample, primers were designed according to the conserved region, and sequencing joints were added to the end of the primers, PCR amplification was performed, and the products were purified, quantified and homogenized to form sequencing libraries. The constructed libraries were first inspected, and qualified libraries were sequenced by Illumina NovaSeq 6000. Raw image data files obtained by high‐throughput sequencing (such as Illumina NovaSeq and other sequencing platforms) were converted into Sequenced Reads by Base Calling analysis, and the results were stored in FASTQ (fq) file format. It contains sequence information of sequencing sequences (Reads) and corresponding sequencing quality information.

### Hemolysis Test

Red blood cells were prepared into 2% suspension with 1 mL of PBS, EcN, EcN_T_, EcN@L, and EcN_T_@L (10^8^ CFU) and of ultrapure water, and the incubated at 37 °C for 1 h. Then the cells were centrifugated at 8000 rpm, and the absorbance of supernatant was measured at 578 nm. Hemolytic ratio was calculated.

### In Vivo Toxicity Assessment

Mice were randomly grouped in two groups (*n* = 5). 200 µL of PBS or 1 × 10^9^ CFU of EcN_T_@L was oral administrated to the mice every other day. Tissues and blood were collected at day 10 post administration. Blood samples from euthanized mice were analyzed by using hematology analyzer. In addition, samples from the colon, ileum, jejunum, duodenum, and stomach were collected for histological analysis and viewed by microscope imaging after staining with HE.

### Statistical Analysis

The data were expressed as average ± SEM. Statistical analysis was performed by using GraphPad Prism and Excel 2016. Differences between the two groups were assessed using the two‐tailed Student *t*‐test, while differences between the multiple groups were assessed using one‐way analysis of variance (ANOVA).

## Conflict of Interest

The authors declare no conflict of interest.

## Supporting information



Supporting Information

## Data Availability

The data that support the findings of this study are available from the corresponding author upon reasonable request.
