# Supplementary material for: Engineered Probiotics Mitigate Gut Barrier Dysfunction Induced by Nanoplastics
Source: Adv Sci (Weinh). 2025 Apr 1;12(22):2417283. doi: 10.1002/advs.202417283 (PMC12165037; doi:10.1002/advs.202417283)
Supplement: Supplementary file 1 — Supporting Information [file ADVS-12-2417283-s001.docx]

Supporting Information

**Engineered Probiotics Mitigate Gut Barrier Dysfunction Induced by Nanoplastics**

Wenxin Chen, Qiyan Guo, Hong Li, Xue Chi, Xiang Ma, Yanqiong Tang, Quanfeng Liang, Zhu Liu, Yong Liu ^*^, Juanjuan Li ^*^

J.Li

School of Life and Health Sciences, Hainan Province Key Laboratory of One Health, Collaborative Innovation Center of One Health, Hainan University

Haikou 570228, China

E-mail: [lijuanjuan@hainanu.edu.cn](mailto:lijuanjuan@hainanu.edu.cn)

Y.Liu

School of Chemistry and Chemical Engineering, Hainan University

Haikou 570228, China.

E-mail: [liuyong@hainanu.edu.cn](mailto:liuyong@hainanu.edu.cn)

W.Chen, Q.Guo, X.Chi, X.Ma,Y.
School of Life and Health Sciences, Hainan Province Key Laboratory of One Health, Collaborative Innovation Center of One Health, Hainan University,
Haikou 570228, China.
Z.Liu
Faculty of Animal Science and Technology, Key Laboratory of Animal Nutrition and Feed Science of Yunnan Province, Yunnan Agricultural University,

Kunming, 650201, China.

Q.Liang
State Key Laboratory of Microbial Technology, Shandong University,

Jinan 250100, China.


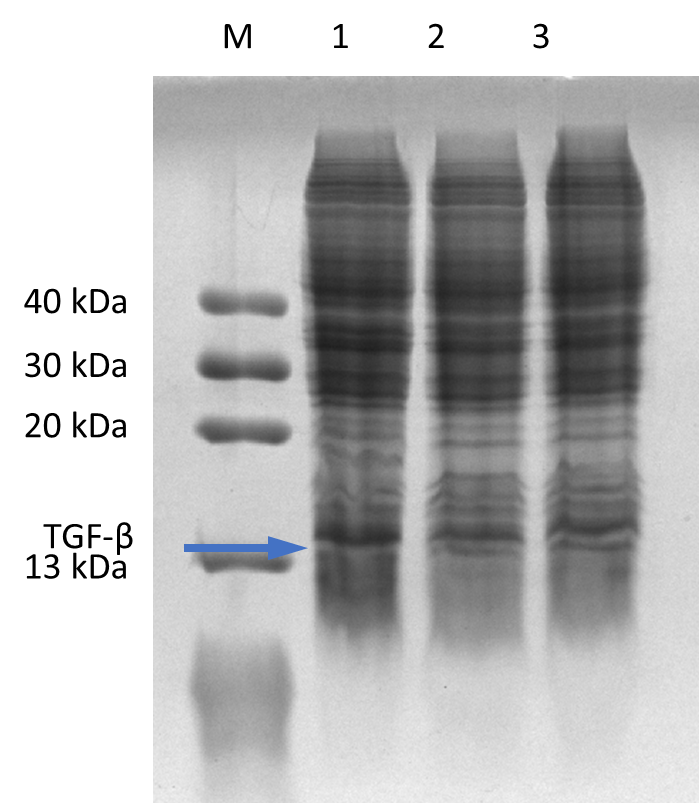


**Figure S1.** The SDS-PAGE analysis of EcN, EcN_T_ and EcN_T_@L. Lane M was marker, lane 1 to lane 3 are EcN, EcN_T_, and EcN_T_@L, respectively.


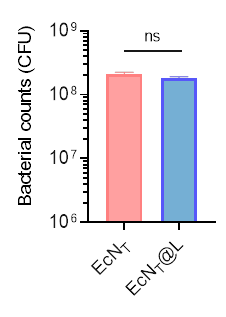


**Figure S2.** Bacterial counts of EcN_T_@L and EcN_T_.


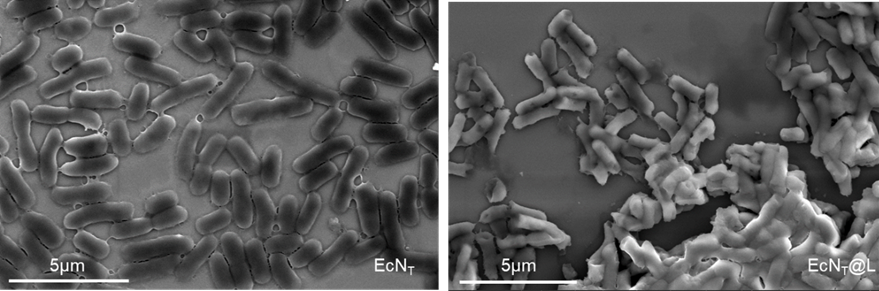


**Figure S3.** SEM images of EcN_T_ and EcN_T_@L. Scale bar: 5 µm.


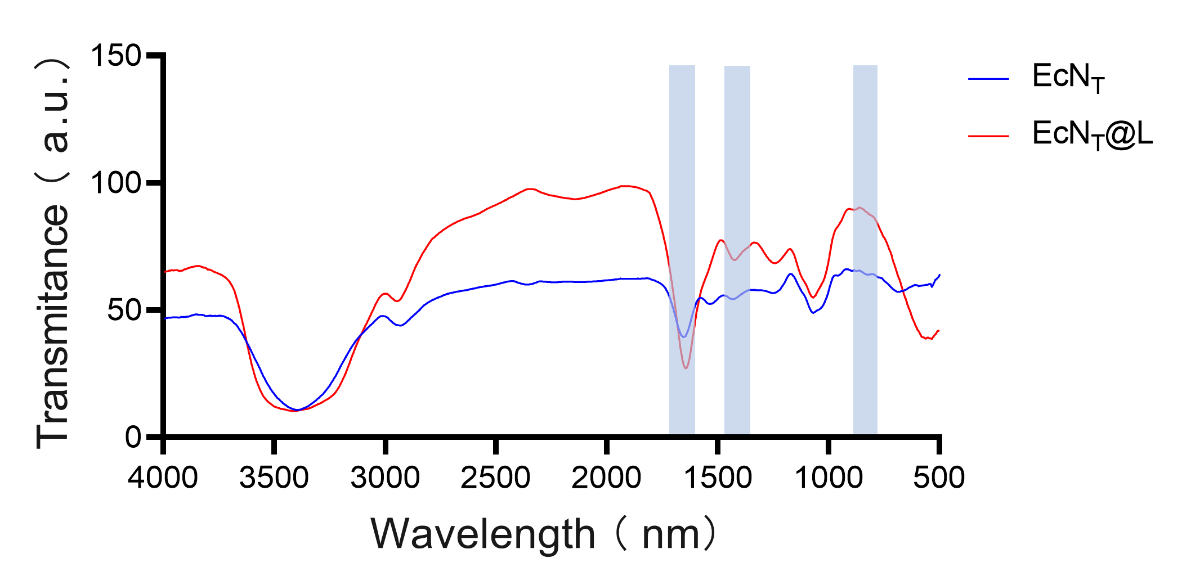


**Figure S4** FTIR spectra of EcN_T_ and EcN_T_@L.

**
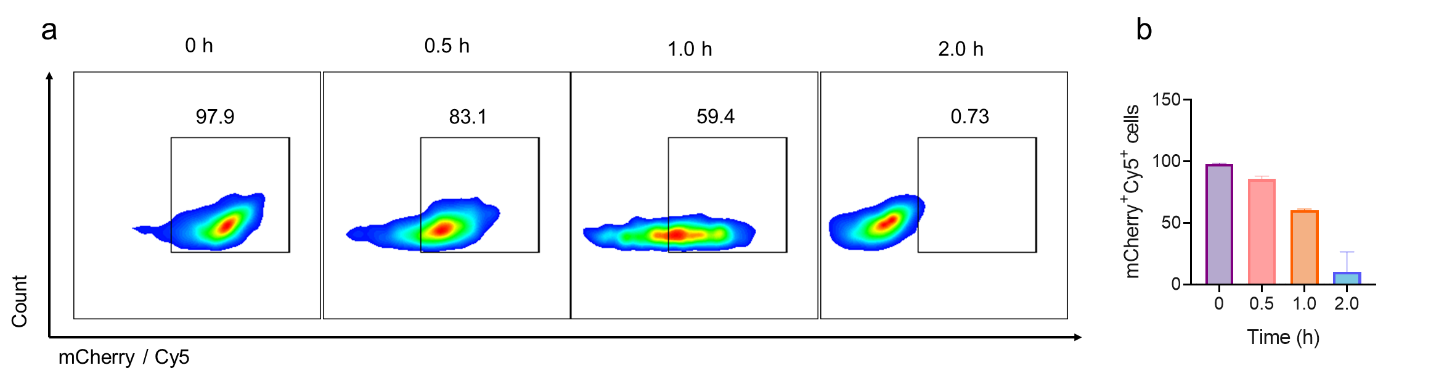
**

**Figure S5** Flow cytometry assay (**a**) and the level of CY5^+^ cells (**b**) of EcN_m_@L-CY5 in SIF post incubation for curtained time point.


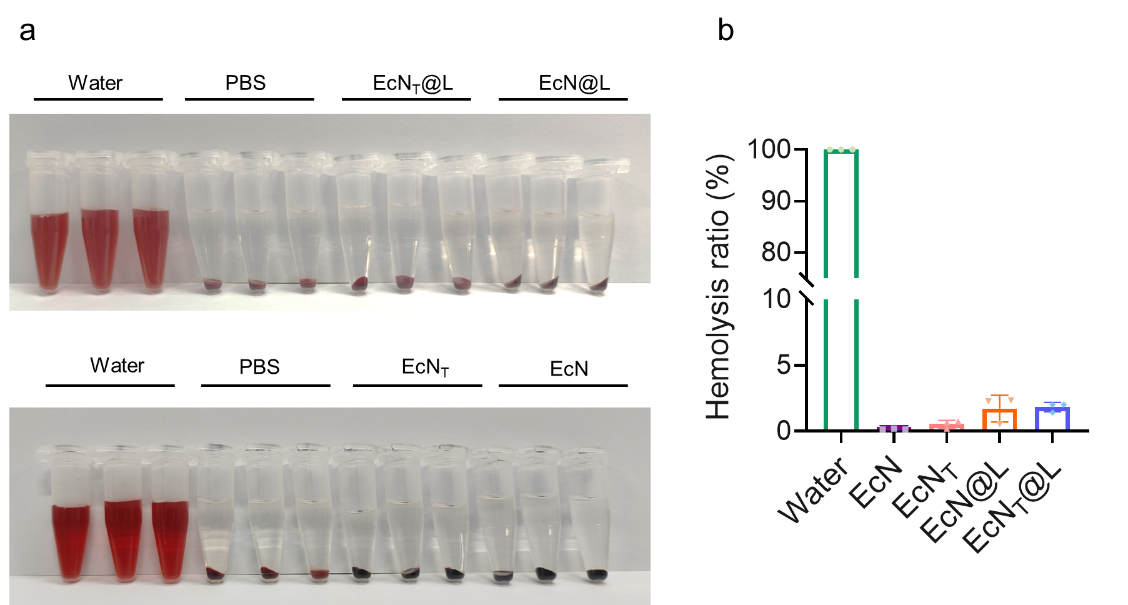


**Figure S6** (**a)** Photographs of blood cells treated with water, PBS, EcN, EcN_T_, EcN@L and EcN_T_@L. (**b)** The hemolysis ratio of water, PBS, EcN, EcN_T_, EcN@L and EcN_T_@L.


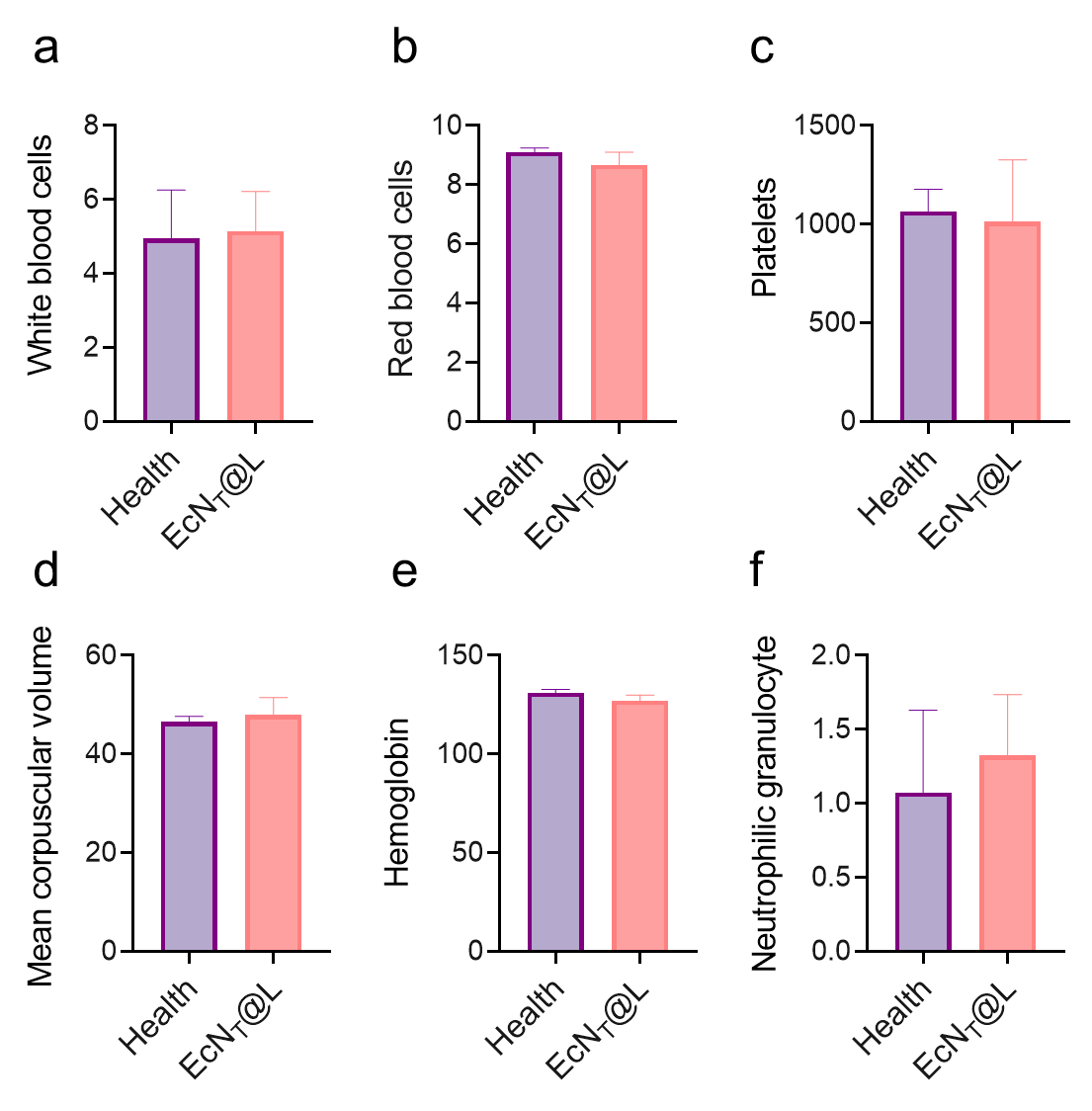


**Figure S7** Counts of white blood cells (**a**), red blood cells (**b**), hemoglobin (**c**), mean corpuscular volume (**d**), Hemoglobin (**e**) and neutrophilic granulocyte (**f**) of mice treated with EcN_T_@L for 10 days.


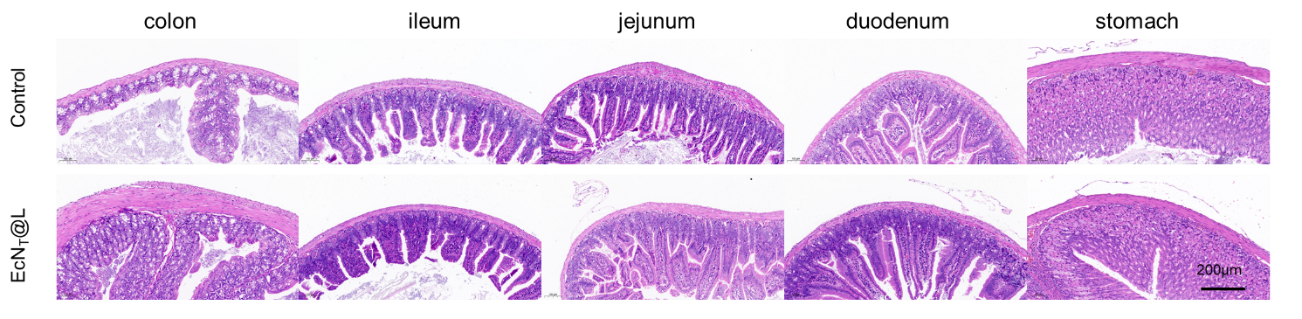


**Figure S8.** HE staining of the colon, ileum, jejunum, duodenum and stomach sections of mice post a 10-day oral administration of EcN_T_@L. Scale bar: 200 μm.


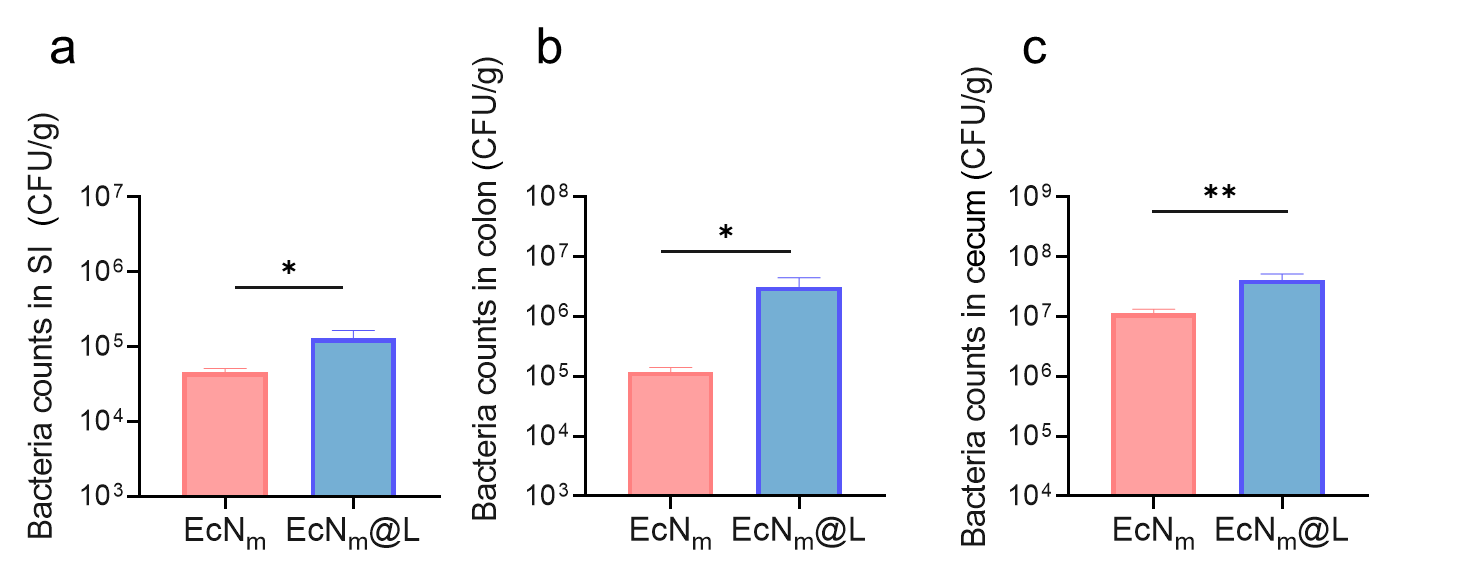


**Figure S9.** Bacterial counts of EcN_m_ and EcN_m_@L in small intestine (a), colon (b) and cecum (c). *n* = 3, **p* ≤ 0.05, ***p* ≤ 0.01.


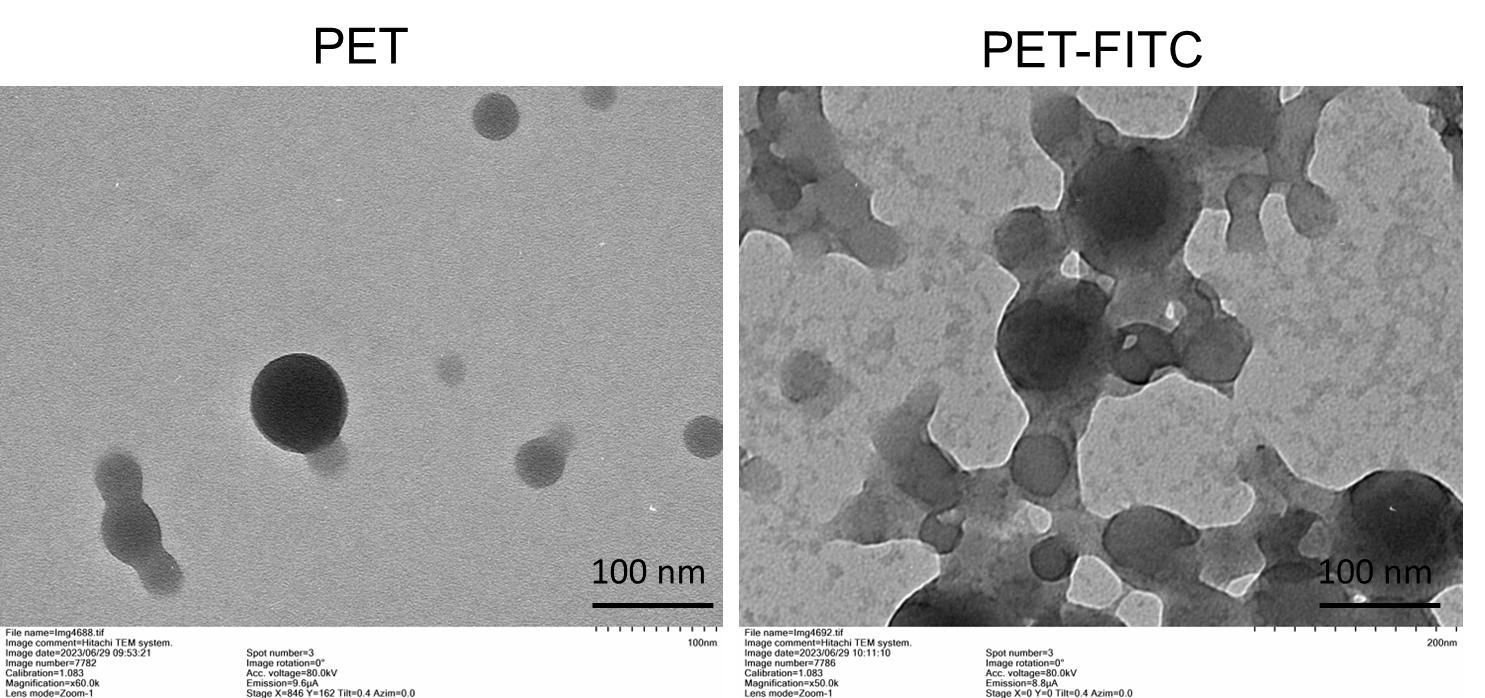


**Figure S10** TEM images of nano PET (a) and Nano PET-FITC (b). Scale: 100 nm


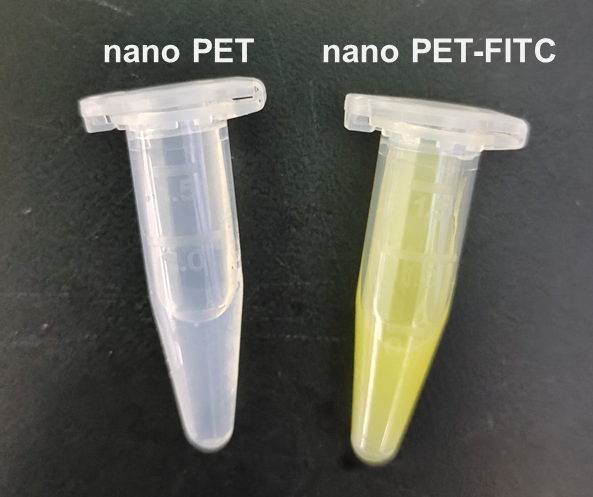


**Figure S11** Photographs of nano PET dispersion and nano PET-FITC dispersion.


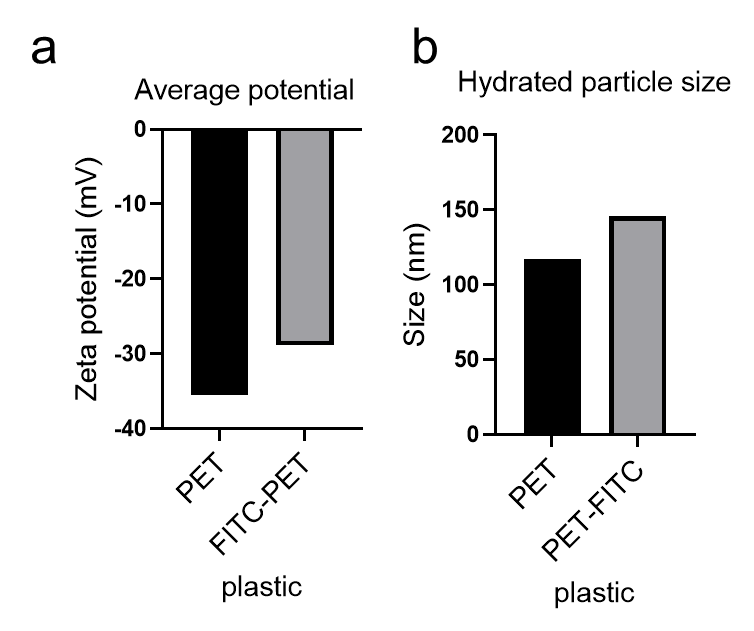


**Figure S12** The (a) ζ-potential and (b) size distribution of nano PET and nano PET-FITC.

**Figure S13.** Relative fluorescence intensity in the substrate of single-layer Caco-2 cell model treated with nano PET-FITC. *n* = 3, **p* ≤ 0.05


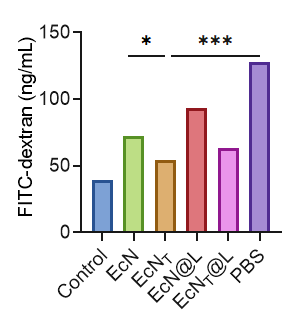


**Figure S14.** The levels of FITC-dextran in the sublayer of nano PET exposed monolayer cell model after treatment with EcN, EcN_T_, EcN@L, EcN_T_@L and PBS. *n* = 3, **p* ≤ 0.05, ****p* ≤ 0.001.

**Figure S15.** Survival rate Caco-2 cells treated with EcN, EcN_T_, EcN@L and EcN_T_@L.


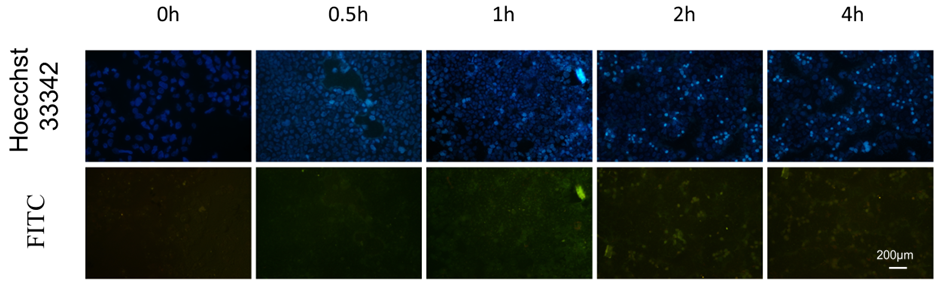


**Figure S16.** Fluorescence images of Caco-2 treated with nano PET-FITC. Scale bar: 200 μm.


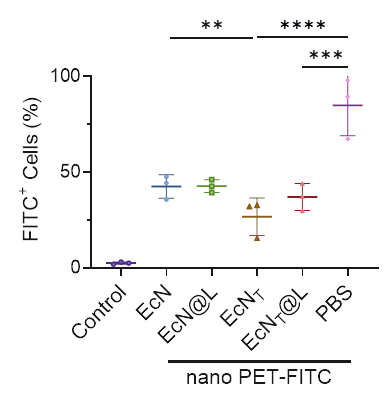


**Figure S17.** The level of FITC^+^ cells post treatment with EcN, EcN_T_, EcN@L and EcN_T_@L and nano PET-FITC. *n* = 3, ***p* ≤ 0.01, ****p* ≤ 0.001, *****p* ≤ 0.0001.

**
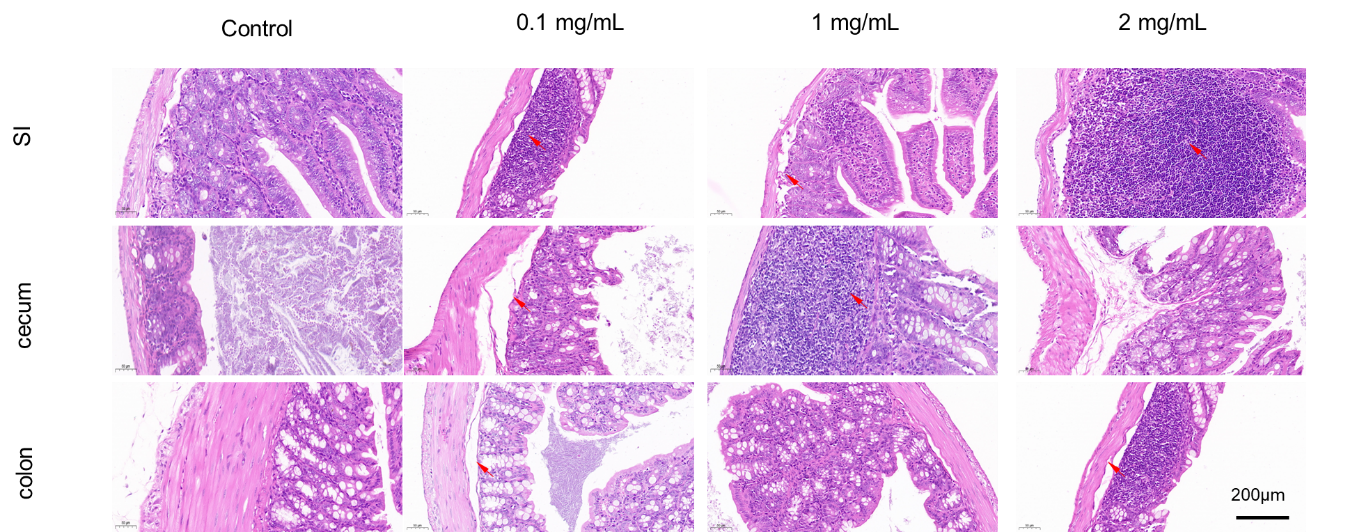
 Figure S18.** HE staining of SI, cecum, and colon of mice post a 28-days oral administration of different concentrations of nano PET. Scale bar: 200 μm.

**
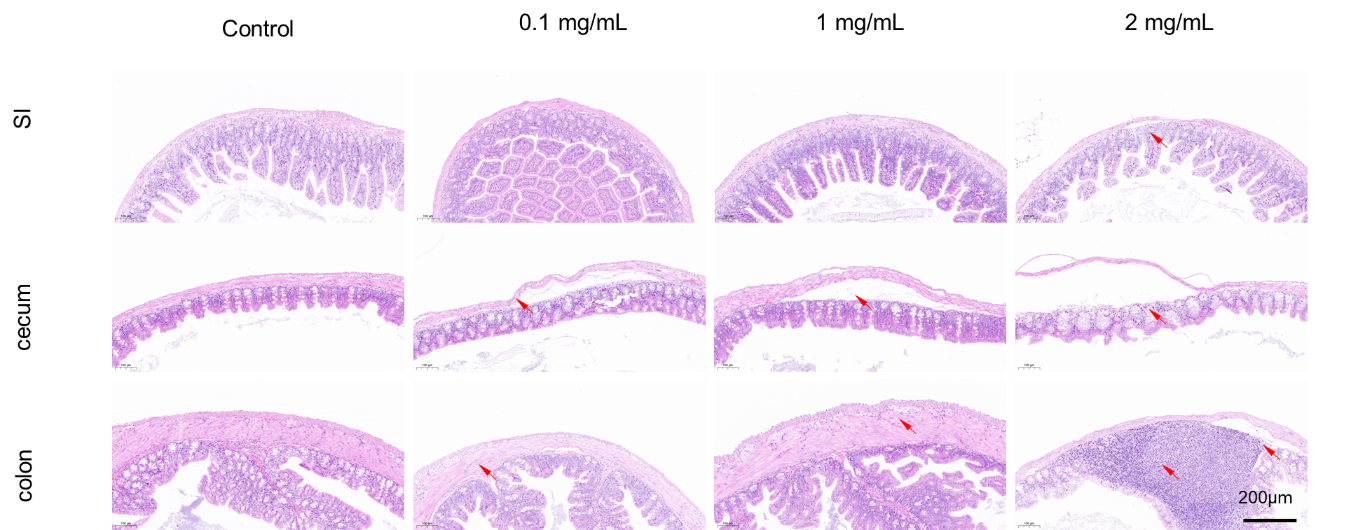
**

**Figure S19.** HE staining of SI, cecum, and colon of mice post a 7-days oral administration of different concentrations of PET. Scale bar: 200 μm.


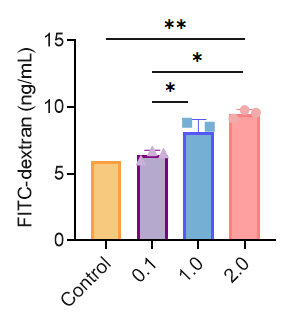


**Figure S20.** The level of FITC-dextran in serum of mice post oral administration of different concentrations of nano-PET for 7 days. *n* = 3, **p* ≤ 0.05, ***p* ≤ 0.01.


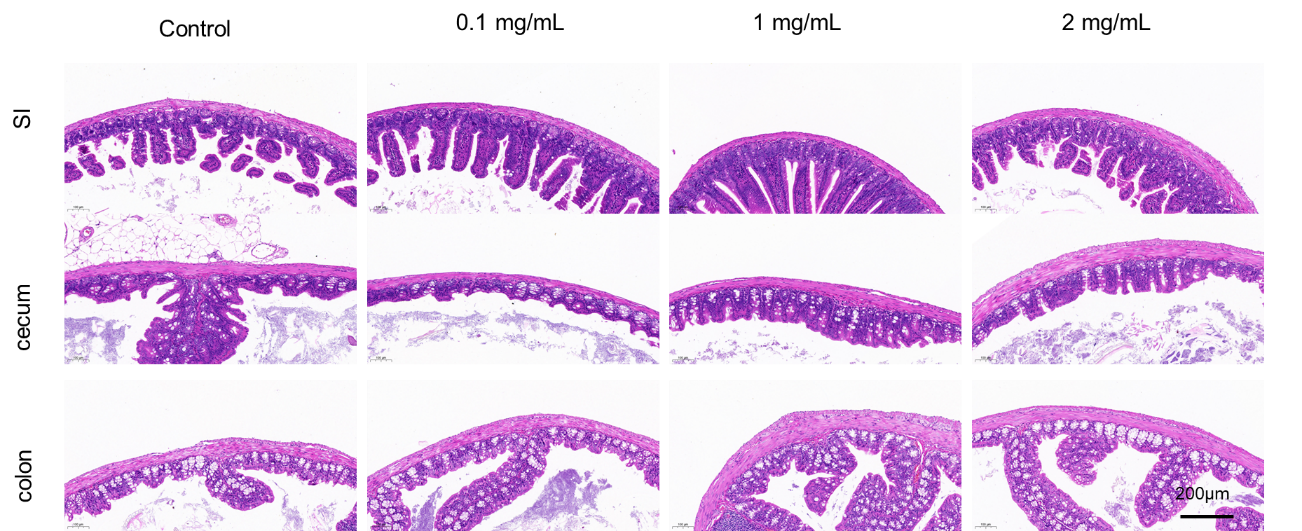


**Figure S21** HE staining of SI, cecum, and colon of mice post a 7-days oral administration of nano PET and a 5-days oral administration of EcN_T_@L. Scale bar: 200 μm.


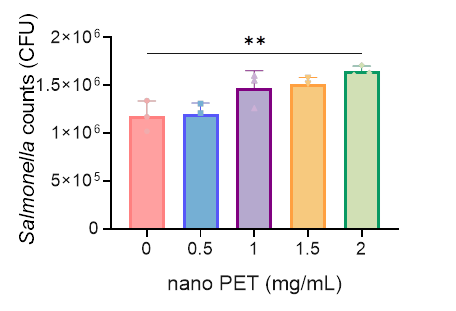


**Figure S22.** The counts of *Salmonella* in cells treated by nano PET (0, 0.5, 1.0, 1.5, 2.0 mg/mL) and *Salmonella* (1 × 10^6^ CFU/mL). *n* = 3, ***p* ≤ 0.01.

**
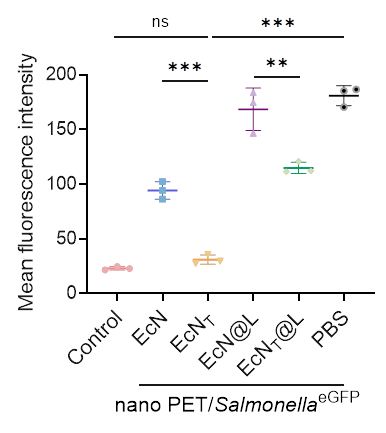
**

**Figure S23**. MFI of cells treated with nano PET/*Salmonella*^eGFP^ and EcN, EcN_T_, EcN@L and EcN_T_@L. *n* = 3, ***p* ≤ 0.01, ****p* ≤ 0.001.

**Figure S24**. Relative expression of iNOS in Caco-2 cells treated with PBS, EcN, EcN_T_, EcN@L, and EcN_T_@L post incubation with nano PET/*Salmonella*. *n* = 3, ****p* ≤ 0.001.

**Figure S25** Relative expression of IL-10 in Caco-2 cells treated with PBS, EcN, EcN_T_, EcN@L, and EcN_T_@L post incubation with nano PET/*Salmonella*. *n* = 3, ***p* ≤ 0.01, ****p* ≤ 0.001.


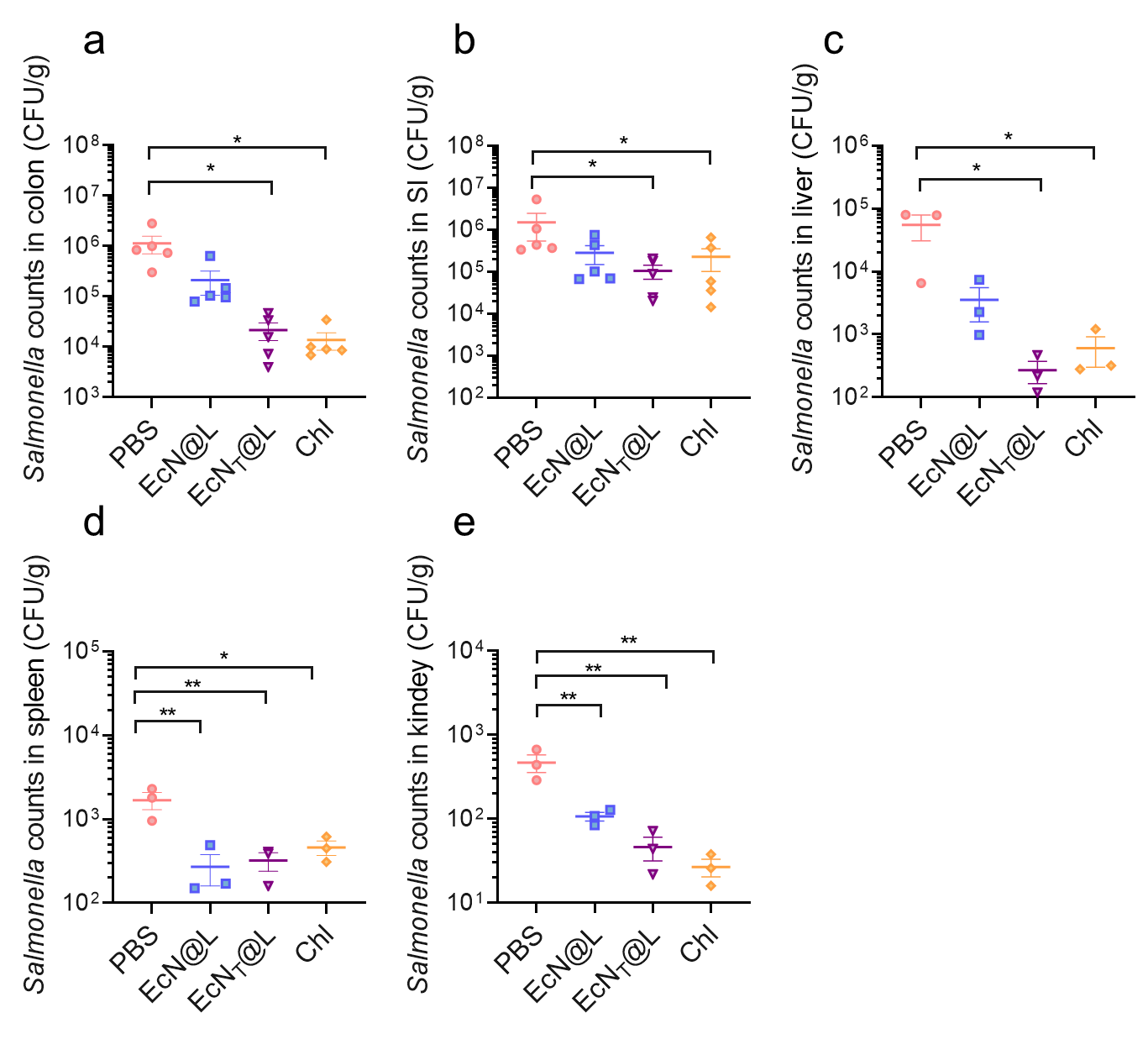


**Figure S26** The *Salmonella* counts in colon (**a**), SI (**b**), liver (**c**), spleen (**d**), and kindey (**e**) in mice post treatment. *n* = 5, **p* ≤ 0.05, ***p* ≤ 0.01.


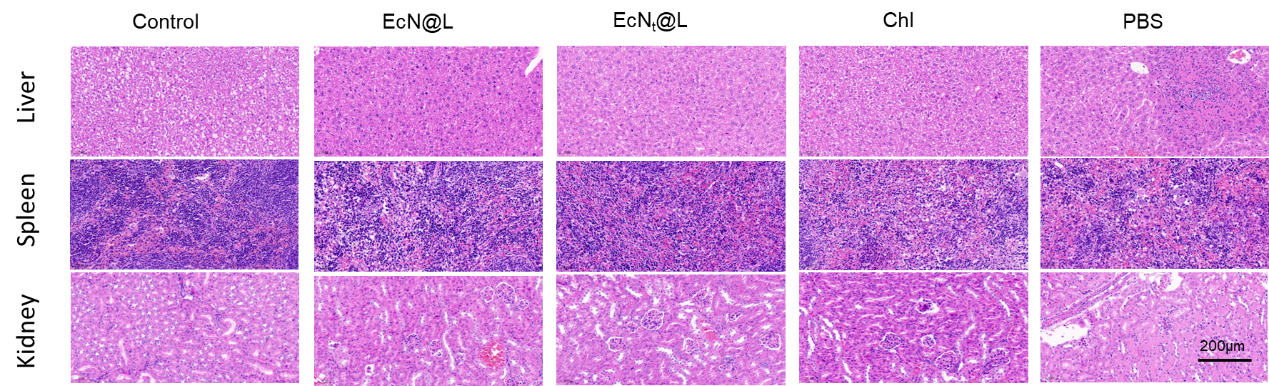


**Figure S27** HE staining of kidney, spleen, liver in mice post treatment. Scale bar: 200 μm.


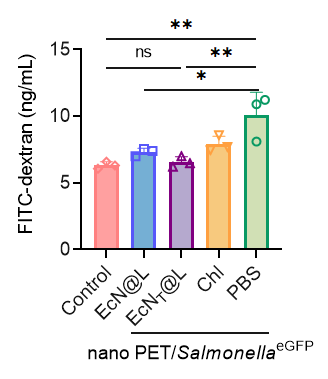


**Figure S28** Levels of FITC-glucan in the blood after treated with PBS, EcN, EcN_T_, EcN@L, and EcN_T_@L post incubation with nano PET/*Salmonella*. *n* = 3, **p* ≤ 0.05, ***p* ≤ 0.01.


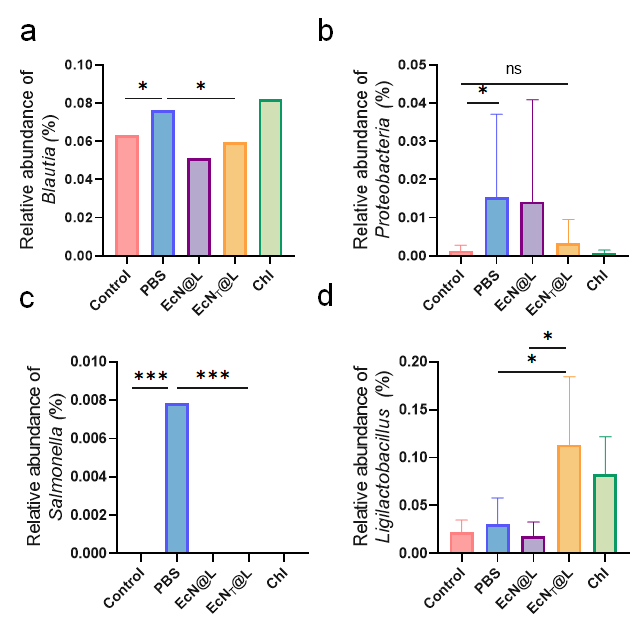


**Figure S29** The relative abundance of *Blautia* (**a**), *Proteobacteria* (**b**), *Salmonella* (**c**), and *Ligilactobacillus* (**d**). *n* = 3, **p* ≤ 0.05, ****p* ≤ 0.001.

**Table S1 Primer sequences.**

| Primer name | Primer sequence F (5’-3’) | Primer sequence R (5’-3’) |
| --- | --- | --- |
| IL-10 | TTTGAATTCCCTGGGTGAGAA | CTCCACTGCCTTGCTCTTATTTTC |
| iNOS | CAACCAGTATTATGGCTCCT | GTGACAGCCCGGTCTTTCCA |
| TGF-β | TGGTGGACCGCAACAACGCC | GGGGGTTCGGGCACTGCTTC |
| IL-1β | AAATACCTGTGGCCTTGGGC | CTTGGGATCCACACTCTCCAG |
| β-actin | CTCTTTGATGTCACGCACGATTTC | GTGGGCCGCCCTAGGCACCAG |
| NFκB(p50) | TGGACCGCTTGGGTAACTCT | CATTCAGACCGTCCCCGTTG |
| RELA(P65) | ATGCTGATGTCGATGCTATG | ATGCTGAAACTGCTCATGCG |
| IKK | ACTCCAAAGTCCGGCAGAAG | CCGTTGGGTATGTGTGAACG |
| IL-6 | GAGGATACCACTCCCAACAGACC | AAGTGCATCATCGTTGTTCATACA |
| TNF-α | TTGACCTCAGCGCTGAGTTG | CCTGTAGCCCACGTCGTAGC |
| IL-8 | ACTGAGAGTGATTGAGAGTGGAC | AACCCTCTGCACCCAGTTTTC |
